# Heuristic pruning of decision trees at low probabilities and probability discounting in sequential planning in young and older adults

**DOI:** 10.1038/s41598-025-00905-7

**Published:** 2025-05-09

**Authors:** Sophia-Helen Sass, Lorenz Gönner, Sarah Schwöbel, Sascha Frölich, Franka Glöckner, Stefan J. Kiebel, Shu-Chen Li, Michael N. Smolka

**Affiliations:** 1 Department of Psychiatry and Psychotherapy, Technische Universität Dresden, Würzburger Str. 35, 01187 Dresden, Germany; 2 Faculty of Psychology, Technische Universität Dresden, Dresden, Germany

**Keywords:** Psychology, Human behaviour

## Abstract

When planning an action sequence, it has been shown that humans prune decision trees to reduce computational complexity, instead of considering all possible options. However, little is understood about pruning employed in probabilistic environments, where actions result in multiple outcomes with varying probabilities, and how decision biases, such as discounting of probabilistic rewards, influence decisions. This study investigates whether participants prune low-probability options in a three-step decision-making task and analyzes the impact of probability discounting on planning. Potential age-related differences in planning strategies are explored in groups of young (aged 18–35 years; n = 57) and older (aged 65–75 years; n = 50) adults. By using reinforcement-learning modeling and model comparison, we show that participants reduce computational demands by pruning decision tree branches of lower probability—a highly efficient strategy in this environment. Additionally, participants reduce their planning depth, i.e., the number of considered steps. Planning is further influenced by discounting high-probability outcomes. Older individuals show stronger reductions in planning depth, an increase in decision noise, and more pronounced probability discounting, which contributes to the observed age-related decline in planning performance. Our findings suggest directions for future research to elucidate the underlying meta-control mechanisms guiding the application of planning strategies.

## Introduction

Planning multiple steps ahead to achieve a goal is a fundamental cognitive ability of humans. In recent years, computational modeling approaches from the field of model-based reinforcement learning (RL) have gained popularity in cognitive neuroscience as a way of describing human planning processes as multi-step decision-making. The model-based RL framework posits that planning arises from an internal model of the environment^1^. This includes knowledge about the situation the planner is in, possible actions and their potential outcomes. Planners use this knowledge to determine the best sequences of actions to reach a desired goal. In this manuscript, when referring to human decision making, we will refer to these decisions about sequences of actions as strategies.

Given the limited cognitive resources of human decision makers, exhaustively evaluating all potential sequences of possible actions and outcomes within a reasonable timeframe can quickly become challenging, if not unfeasible, due to the exponential increase of options with the number of action steps. To address this challenge, multi-step planning experiments in conjunction with model-based RL approaches have been conducted in the past to identify planning strategies employed by humans (see Mattar & Lengyel^2^ for a review). These studies found behavioral and neural evidence for cost-reducing strategies that ignore parts of the information, often referred to as heuristics^3^, instead of an exhaustive and costly evaluation of all possible options^4–9^. One crucial heuristic that has been shown is the pruning of decision tree branches, i.e., the reduction of planning breadth by ignoring branches associated with unpromising options^5,6,8^.

So far, studies on planning strategies primarily focused on deterministic environments, where selected actions cause reliable transitions to expected outcome states^4,6,9–11^. Such prior work has therefore only partially illuminated real-life decision-making, where action-outcome transitions are often probabilistic. However, it is reasonable to assume that human planning in probabilistic environments differs from planning in deterministic contexts. The complexity and computational costs of planning increase when actions can lead to multiple outcomes with different probabilities, rather than a single deterministic outcome. Consequently, even more than in deterministic environments, planners are likely to apply heuristics, i.e. ignoring parts of the decision tree, to reduce complexity. In a previous study addressing planning in a three-step probabilistic environment, we found that participants did not plan all of the three possible steps ahead, i.e., participants limited the number of steps that they considered^12^. In these analyses we assumed full-breadth planning (i.e. evaluating all options at each step), with the only cost-reducing heuristic being a limitation of planning depth. This did not take into account that reducing decision tree complexity in a probabilistic setting could also be based on the state transition probabilities: in scenarios such as our probabilistic planning task^12^, where the outcomes have different probabilities, ignoring branches of the decision tree with low probability, e.g., 5%, results in minimal information loss. Consequently, pruning these branches can save cognitive effort with little impact on the outcome, making it a useful and intuitive heuristic. In the present study, we therefore tested whether the complexity of the decision tree is reduced beyond the variation of the planning depth by pruning branches with low probabilities.

The second aspect we address is how planners discount probabilistic rewards^13^. It has been shown that the subjective value assigned to an outcome progressively diminishes from its nominal value as the odds of obtaining it decrease, reducing its probability of being chosen, while the value of certain outcomes remains unaffected (see Green & Myerson^14^ for an overview). This phenomenon, known as probability discounting, can be mathematically formalized as a hyperbolic discounting function^15^. While this formalization is more commonly associated with temporal discounting—where rewards are subjectively devalued based on their delay—it is equally applicable to probability discounting. Probability discounting causes a bias in decision making as the planner’s environmental model may deviate from the real environment. This influences their evaluation of action sequences and, consequently, their action choices. In the present study, we tested whether participants discount probabilistic outcomes during planning.

Pruning heuristics and probability discounting could further be influenced by individual differences between participants, particularly age given age-related declines in neurocognitive resources^16,17^. However, in the existing body of literature, there is a notable absence of studies that specifically address planning strategies in complex probabilistic environments across the lifespan. Studies suggest an age-related decline in planning performance^18^, associated with limitations in cognitive resources like working memory and processing speed in older adults as well as reduced reward sensitivity and inefficient decision strategy shifting together with deficits in representing and updating reward values^16,19–21^. In probabilistic environments, multiple options may increase the importance of cost-reducing pruning heuristics, especially for older individuals. We previously reported a stronger reduction in planning depth as a cost-saving pruning heuristic in older adults compared to younger ones^12^, which could explain differences in planning performance. It has also been discussed that the increased cognitive resources required to process probabilistic options induces risk-avoidant behavior in older individuals^22–24^, resulting in suboptimal performances. Aging also influences the valuation of certain versus uncertain outcomes (see Frank & Seaman^25^ for a review). Further findings indicate that, in comparison to young adults, older individuals show stronger risk-averse behavior for gains, i.e., stronger preference for certain gains, and a stronger risk-seeking behavior for losses, i.e., a stronger avoidance of certain losses^22,26,27^. Hence, older individuals may differ from young individuals in their subjective valuation of risky and certain options in a multi-step planning process and therefore show a discounting bias for probabilistic outcomes that differs from young planners.

In this study, we employ a revised version of our probabilistic planning task (adapted from Steffen et al.^12^) to address three key questions: first, do participants reduce the complexity of the decision tree by pruning lower probability branches? Second, do participants discount probabilistic outcomes when planning? Lastly, does age influences both decision tree pruning and the discounting of probabilistic outcomes?

## Methods and materials

### Participants and study procedures

We collected complete datasets of 60 young adults (18–35 years) and 57 older adults (65–75 years). Participants were screened for potential exclusion criteria because of present substance use disorders and other psychiatric or neurological disorders. Further exclusion criteria for both groups were current dopamine-agonistic medication, severe and uncorrectable constraints in eyesight, and motor impairments of the hands and fingers that constraint response behavior. Older adults were additionally screened for mild cognitive impairment using the German version of the Montreal Cognitive Assessment version 8.1 (MoCa)^28^ adapted for administration by telephone, applying a cut-off score of 18 out of 22 points^29^. Participants gave written informed consent prior to the study inclusion interview and assessment. They were told they will receive 25–35 Euro as compensation, depending on their planning task performance. The study was approved by the ethics committee (EK536122019) of the TUD Dresden University of Technology and performed in accordance with relevant guidelines and regulations.

The experiment was conducted as a browser-based online study. Participants used their private desktop computers, laptops or tablets with external keyboards and performed the experiment at a time of their choice. The session took 2.5–3 h including optional breaks. Participants went through sociodemographic and psychological questionnaires, the probabilistic planning task, and a neuropsychological test battery in a fixed sequence. Prior to all analyses we excluded participants who performed below chance level in the probabilistic planning task. This criterion led to the exclusion of three young and seven older adults, resulting in a final sample of 57 young and 50 older adults. Demographic characteristics and (neuro-) psychological measures of the final sample are reported in Table 1 (see Results). Groups did not differ in gender distribution (*χ*^2^(1) = 1.41, p = 0.235). Significantly more young participants, relative to older participants, had a higher education degree (*χ*^2^(1) = 5.35, p = 0.021). To control for potential operational confounds, we assessed the frequency of using electronic devices such as smartphones or (tablet) computers, and computer/video gaming. All participants reported a regular use of electronic devices, i.e., at least once per week. Age groups did not differ in frequency of regular computer/video gaming (*χ*^2^(1) = 0.01, p = 0.946).

**Table 1:** Descriptive statistics and group comparison of outcomes of planning task (SAT) and neuropsychological task battery.

	Young adults (*n* = 57)	Older adults (*n* = 50)	Test statistic	*p*
Sample characteristics & questionnaire data				
Age	26.33 (4.50)	69.14 (2.73)	–	–
Gender (F/M)	30/27	32/18	1.41^c^	.235
Higher education (%)^a^	89.47	72.00	5.35^c^	**.021**
Regular^b^ use of electronic devices (%)	100.00	100.00	–	–
Regular^b^ PC/video gaming (%)	24.56	24.00	0.01^c^	.946
Need for cognition	12.63 (13.32)	19.48 (12.88)	− 2.69^d^	**.008**
Neuropsychological task performances (%)				
Spot-a-word	63.99 (10.98)	77.33 (8.96)	− 6.82^d^	**< .001**
Spatial working memory	89.48 (8.17)	79.43 (9.11)	6.02^d^	**< .001**
Identical pictures	69.83 (11.76)	48.57 (7.33)	11.36^f^	**< .001**
Neuropsychological task response times (s)				
Spot-a-word	3.90 (1.08)	4.17 (1.52)	− 1.07^d^	.29
Spatial working memory	1.10 (0.27)	1.74 (0.38)	2630^g^	**< .001**
Identical pictures	2.31 (0.45)	3.55 (0.73)	2723^g^	**< .001**
SAT relative performance (%)				
Low noise	85.91 (11.68)	69.89 (12.46)	^h^	
High noise	83.13 (14.70)	77.02 (14.39)		
SAT planning time (s)				
Low noise	8.56 (5.28)	8.47 (5.04)	^h^	
High noise	7.59 (3.99)	8.06 (4.46)		

Scores represent means and standard deviations (in parenthesis). Task performance indicated percentage of correct responses within the time limit.

Significant values are in bold.

^a^≥ 12 years of school education.

^b^At least once per week.

^c^Pearson’s chi-squared test with one degree of freedom and corresponding p-value.

^d^Standard two-sample t-test with 105 degrees of freedom.

^e^Group- and condition-wise means and standard deviations were compared in a mixed ANOVA model.

^f^Welch’s t-test with 95.2 degrees of freedom.

^g^Wilcoxon rank sum test.

^h^Two-way mixed ANOVA results reported in section “behavioral measures”.

### Psychological and cognitive measures

For group comparisons in all psychological and cognitive measures that were used for sample description, the data distribution was first tested for normality and homoscedasticity. Statistical tests were applied accordingly (see Table 1).

To assess the participants’ tendency to readily engage in thinking as a potential covariate for probabilistic forward planning, we used the German 16-item-version of the Need for Cognition Scale (NFC, Bless et al.^30^). In our sample, older adults scored higher in the NFC compared to younger adults (t(105) = − 2.69, p = 0.008). To assess basic cognitive abilities, we used a small selection of neuropsychological tasks as those used in the Berlin Aging Study^31^ (see Steffen et al.^12^ for detailed task descriptions). Processing speed as correlate of fluid intelligence was measured with a picture matching task (Identical Pictures Task, IDP). The examination of verbal knowledge as correlate of crystallized intelligence was based on a German vocabulary test (Spot-a-Word Test; SAW). We assessed spatial and serial working memory using the Spatial Working Memory Task (SWM)^32,33^. In the SWM, we evaluated spatial location and serial order working memory for low and high working memory load conditions across four subtasks. Since we did not obtain enough reliable data from the serial memory subtask due to potential misunderstandings of the task instruction, we only included data of the spatial memory subtask in our analyses. In IDP, SAW, and SWM, accuracy (percentage of correct responses) and response times were acquired. IDP accuracy as the ratio of correct responses, given within the task’s time limit of 80 s, to the total number of items was used as an indicator of processing speed. For all cognitive measures, trials with response times below 150 ms were excluded, since this is a common timeframe for solely perceptual and motor processes^34^. All participants gave at least 10 correct responses per task measure.

Our results for neuropsychological task performances (Table 1) were in line with previous findings^16,33,35^: older adults outperformed young adults in the SAW as a measure of crystallized intelligence (t(105) = − 6.82, p < 0.001) and showed a reduced performance in measures of fluid intelligence in the IDP (t(105) = 11.32, p < 0.001) and SWM (t(105) = 6.02, p < 0.001).

Additionally, we assessed reasoning using a short, 12-item form of Raven’s Advanced Progressive Matrices (Raven APM)^36^ at the end of the testing session. Older adults showed a lower performance than young adults (t(105) = 8.027, *p* < 0.001). In total, 17 older adults gave less than two correct responses. Altogether 16 of them completed the task in less than a third of the 15-min time limit. Given normal performance in other measures, motivational issues or task misunderstanding may have led the respective participants to prioritize speed over accuracy. Due to this potential confound, we excluded the Raven APM from further analyses.

### Probabilistic planning task

**Fig. 1:**
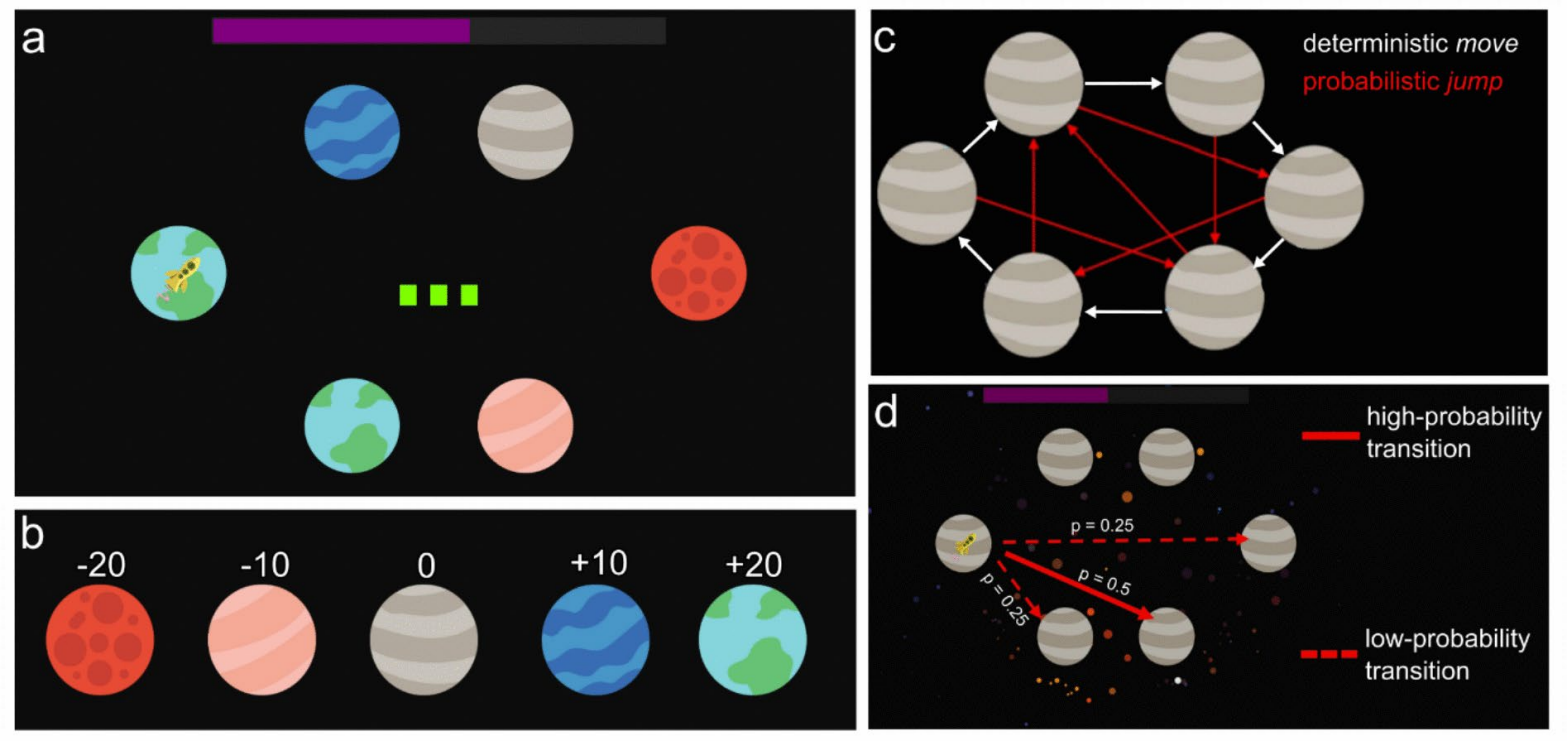
Schematic of the space adventure task. (**a**) Example mini-block with three action steps (green squares) and low noise (black background). There are six planets with the yellow rocket on the left planet, indicating the current location. The fuel bar at the top of the screen showed the accumulated amount of fuel points throughout the task. (**b**) The five planet types with their respective gain or loss of fuel points. (**c**) Transition matrix of the deterministic move action (white arrows) and the probabilistic jump action (red arrows). The matrix was once presented to participants for memorization and practiced during training. (**d**) Visualization of outcome uncertainty for the probabilistic jump action to the target planet (solid arrow, high-probability transition) and its neighbors (dashed arrows, low-probability transitions). Asteroids in the background indicate high-noise condition, where transitions probabilities were 50% (high-probability transition) and 25% (respective two low-probability transitions). In the low-noise condition, transition probabilities were 90% (high-probability transition) and 5% (respective two low-probability transitions). Participants were informed about probabilistic transitions and potential outcomes in general but had to infer the probabilities from experience in 10 respective training mini-blocks.

The Space Adventure Task^12^ (SAT; https://github.com/expfactory-experiments/space_adventure_pd) is a probabilistic multi-step decision-making task that requires forward planning of action sequences in order to maximize reward. The basic task principle was adapted from previously published deterministic planning tasks^5,37^. Participants navigated a spaceship and visited a sequence of three planets in a planetary system (Fig. 1a). A planetary system was a constellation of six planets of five possible planet types (Fig. 1b). The different planet types either cost or added game points (fuel) to the spaceship. The goal was to accumulate the maximum amount of fuel throughout a sequence of 140 varying planetary systems (mini-blocks) and starting positions of the spaceship. A bar at the top of the screen indicated the current amount of accumulated fuel. In each mini-block, participants had three action steps to maneuver the spaceship through the planetary system. The number of remaining action steps were shown in the center of the screen. In summary: In every mini-block participants were presented with a new planetary constellation and starting position of their spaceship. They had three action steps to move the spaceship to different planets to collect fuel points. Participants had to plan forward to find the optimal sequence of action steps leading to the maximum possible fuel gain.

At each action step, participants chose between two actions to maneuver the spaceship: Either *move* clockwise to the neighboring planet or *jump* to a specific non-neighboring planet following a predefined transition pattern (Fig. 1c). *Move* was a deterministic action with 100% certainty, while *jump* involved uncertainty regarding the outcome of the transition. For the *jump* action, there were three possible outcomes: The target planet as the outcome with a high transition probability of 90%, or 50% respectively, and two outcomes with lower transition probabilities of 5%, or 25% respectively. In case of a low-probability transition (5% or 25%), *jump* led to one of the neighboring planets of the target planet, each with equal probability (Fig. 1d). Transition uncertainty was manipulated in two conditions. In the low-noise condition, the high-probability transition had a 90% probability, while in the high-noise condition (cued by asteroids as colored dots in the background, see Fig. 1d) it had only a 50% probability. Jumping to one specific neighboring planet was less probable (low noise: 5% for each neighboring planet; high noise: 25% for each neighboring planet). The state transition probabilities of a *jump* action depended exclusively on the noise condition, cued with the presence or absence of the asteroids in the background. They did not change with the state the rocket was in. The noise conditions were altered every three to six mini-blocks in a pseudo-randomized order, resulting in a total of 70 high and 70 low noise mini-blocks. The high-noise condition was cued asteroids in the background of the screen (colored dots, see Fig. 1d). Action choices and planning time (duration between stimulus onset and button press for first action choice) were measured for each mini-block.

Prior to the experiment, participants were trained on the goal, transition matrix and noise conditions of the task. They were informed about the probabilistic nature of the *jump* transition, without being given the respective probabilities explicitly. Instead, they were expected to learn those probabilities during dedicated training mini-blocks. Participants were instructed to look for the optimal sequence of actions in each mini-block in order to maximize their reward. They underwent specific training mini-blocks including feedback about their route choices. The experiment was controlled using the desktop computer keyboard or external tablet keyboard. *Move* or *jump* actions were selected with the ‘Y’ key and the ‘M’ key on the German (QWERTZ) keyboard using the left and right index finger, respectively. The assignment of the actions to the keys was counterbalanced among the participants.

After participants had completed the SAT, we re-assessed their understanding of task goals, consequences of transition uncertainty, and their knowledge of the *jump* transition pattern with a short debriefing questionnaire. This questionnaire was implemented to filter out potentially confounded data due to false assumptions about the basic task rules (Fig. S1).

### Cognitive models

#### Overview of cognitive models

To assess possible planning strategies, we implemented four computational RL models to fit the participants’ data. Each model is a mathematical formulation of one of the hypothesized strategies and biases underlying the participants’ action choices in the task (full-breadth planning, discounted full-breadth planning, low-probability pruning, discounted low-probability pruning). We compared the fit of the four models and used model comparison using the Bayesian Information Criterion (BIC) to test for which strategy we find the highest evidence. All cognitive models refer to a strategy and/or bias within one action step in the sequence. Additionally, all models allow us to infer the depth of planning, i.e. how many action steps were considered by the participants given the respective strategy or bias.

**Fig. 2:**
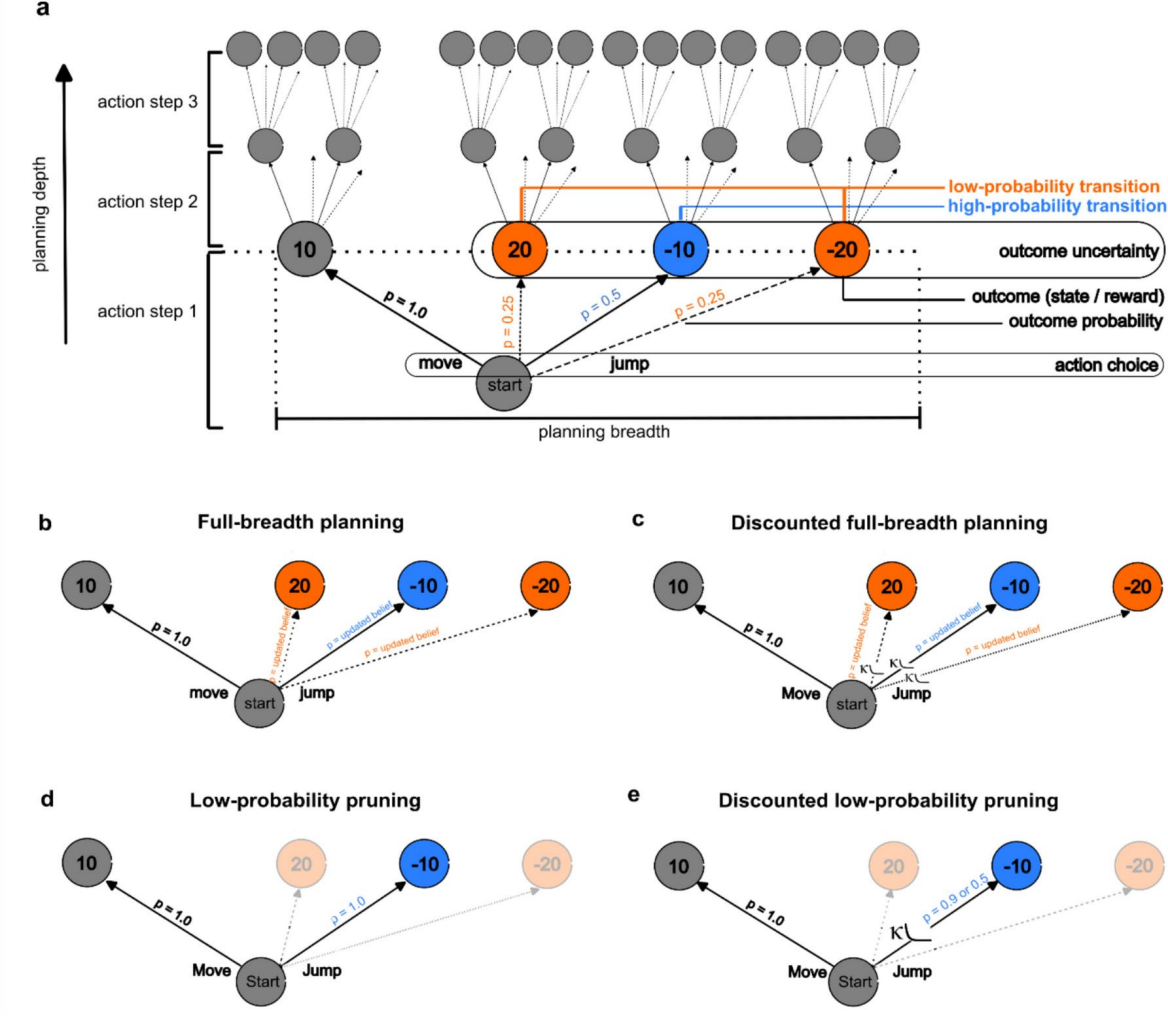
Schematic of the decision tree in the SAT and the four alternative planning strategies. (**a**) The schematic refers to the example mini-block shown in Fig. 1a. Branches following low-probability transitions from action step 2 onwards are not shown for clarity. (**b**–**e**) Each of the four figures refers to the decision tree for one action step in the SAT according to each decision strategy for the four cognitive models. The schematic refers to action step 1 in the example mini-block shown in Fig. 1a and is generalized for both, the low-noise condition (p = 5% low-probability transition) and the high-noise condition (p = 25% low-probability transition). The orange planets indicate the low-probability transitions; the blue planet indicates the high-probability transition according to the transition matrix. (**b**) Full-breadth planning: All possible outcomes and their learned probabilities are considered during planning. (**c**) Discounted full-breadth planning: All possible outcomes and their learned probabilities are considered during planning. Probabilistic outcomes are discounted with a participant-specific discounting factor (κ) based on their learned probabilities. (**d**) Low-probability pruning: Low-probability outcomes are pruned. Only high-probability outcomes are considered during planning. All transitions are treated as deterministic. (**e**) Discounted low-probability pruning: Low-probability outcomes are pruned. Only high-probability outcomes are considered during planning. They are discounted with a participant-specific discounting factor (κ) based on their real transition probabilities.

We will first provide an overview of the assumptions of the four planning models that were compared, see also Fig. 2. In the next section, this is followed by a mathematical description of the models with all the details. The models differ in how many of the possible options are considered at each planning step, if their respective probabilities are taken into account, and how these probabilistic outcomes are (subjectively) valuated. The *full-breadth planning* model (Fig. 2b) assumes that the decision tree is assessed at full breadth for each action step, considering experience-based beliefs about probabilistic contingencies in state transitions. With the *Low-probability pruning* model (Fig. 2d) we propose a cost-reducing heuristic that involves ignoring low-probability transitions for *jump* actions and treating high-probability transitions as deterministic. By pruning low-probability branches of the decision tree, the planning breadth is reduced from four to two branches per action step, essentially creating a deterministic state-transition structure. We also propose biased alternatives for both models, incorporating probability discounting (Fig. 2c,e). Probability-dependent subjective values for probabilistic *jump* actions, shaped by the individual hyperbolic discounting function (8), are assigned to the planner’s options. This affects action choice behavior depending on how strongly *jumps* will be discounted by the planner. In the discounted full-breadth planning this affects all three possible *jump* outcomes. In the discounted low-probability pruning model the remaining high-probability transition will be considered as probabilistic and discounted. Hence, the effort for planning will be reduced but uncertainty is not ignored and decisions depend on subjective (discounted) rather than objective values of probabilistic outcomes.

**Fig. 3:**
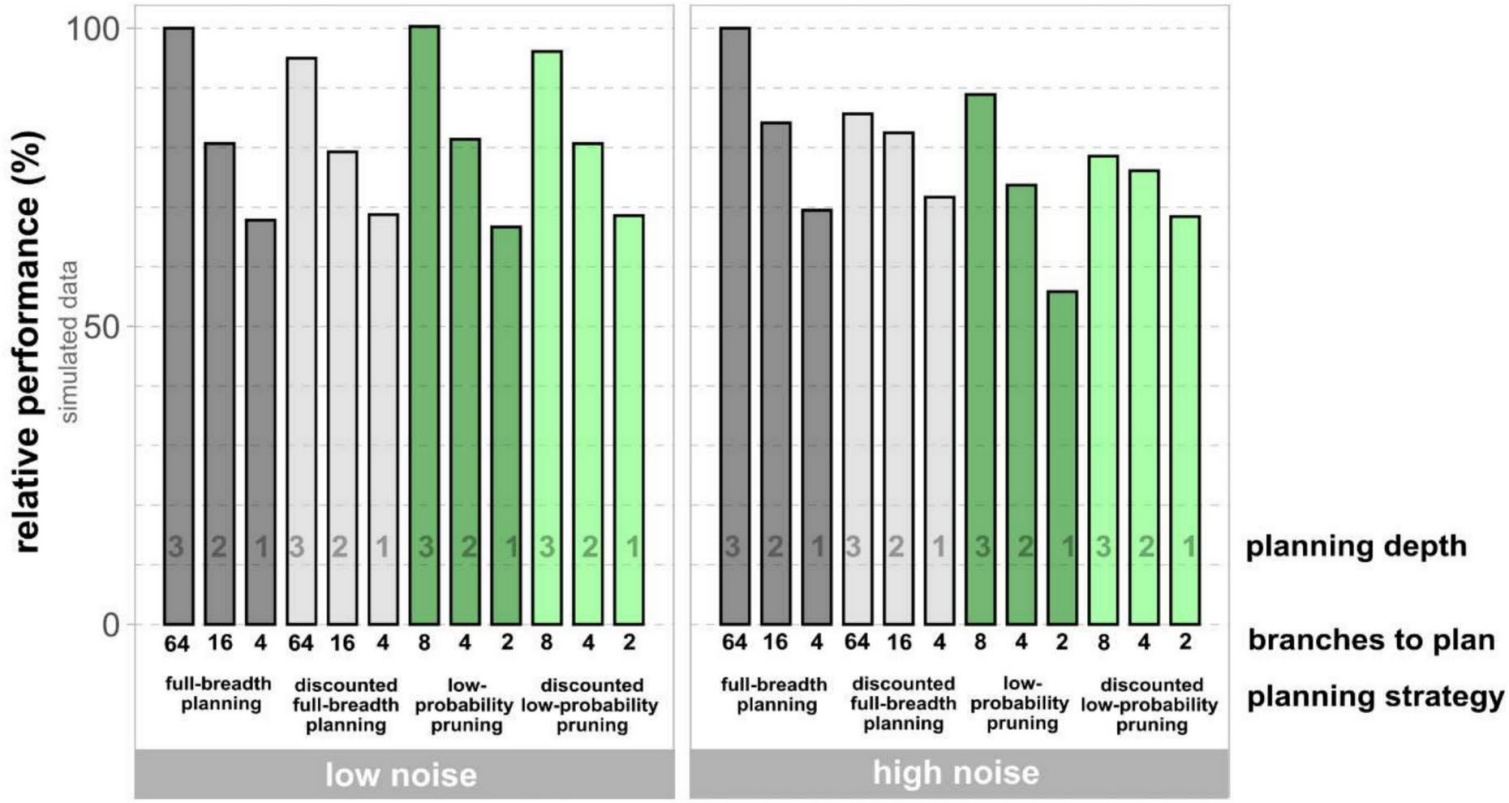
Comparison of points earned in the SAT across 120 mini-blocks for various strategies (simulated data). The relative performance was scaled between optimal performance (full-breadth planning strategy, planning depth three) and random performance as null reference. The relative performance for each strategy is informed by the sum of the average gain of points per mini-block of 1000 agents. The discounting factor for the respective models was set on $\kappa$ = 3 (8).

Even in our three-step planning task full-breadth planning is computationally demanding, because there is one possible outcome planet for the *move* action and three possible outcome planets for the *jump* action (Fig. 2b). In order to plan all steps ahead with each of the four possible outcomes, it is necessary to compute 4^3^ = 64 branches. By limiting the planning depth, the number of choice options and therefore computational costs can be reduced considerably, especially for the third step. However, results from agent simulations show that reducing planning depth will also result in a relatively large decrease in performance, i.e., earned fuel points (Fig. 3). In contrast, compared to full-breath planning, the low-probability pruning strategy is much more efficient in terms of reward earnings per branch to plan (Fig. 3). Low-probability pruning reduces the effort to plan three steps ahead to one-eighth with optimal performance in the low-noise condition, and 89% performance under high noise.

### Mathematical description of cognitive models

The alternative cognitive models were developed based on the default full-breadth planning model that was adapted from Steffen et al.^12^. The code and scripts for performing the model-based inference with the four models are available in the GitHub repository (https://github.com/SophiaHelenSass/SAT_PD2_age).

1. *Full-breadth planning:* In the full-breadth planning model, participants’ action choices per mini-block are modeled in a mixture model of three model-based RL agents. Each agent is implemented with a planning depth $d$ of one, two, or three steps, respectively. The agents’ environmental model includes the available actions $A=\left\{{\prime}mov{e}{\prime},{\prime}jum{p}{\prime}\right\}$ and states $S$ (the planet positions), the transition probabilities $p\left({s}_{t+1}|{s}_{t},{a}_{t}\right)$ for reaching a subsequent state ${s}_{t+1}$ from a given state ${s}_{t}$ with action ${a}_{t}$, as well as the immediate reward $r\left({s}_{t}\right)$ which is returned upon reaching a state ${s}_{t}$. Agents plan their choices when in a specific state in a specific mini-block $b$, by computing the expected cumulative reward for executing each action (so-called “$Q$-values”) with an optimal forward planning algorithm (value iteration algorithm^1^), which is only limited by an agent’s planning depth. An agent with a planning depth of three evaluates Q-values using all three steps in the mini-block, whereas an agent with planning depth one or two only considers the next one or two steps, respectively. This planning process is repeated at each action step $t$ from the current state ${s}_{t}$.
  The value iteration algorithm with planning depth $d\in [1, 2, 3]$ is mathematically defined as:
  1 $$Q\left({s}_{t},{a}_{t},d\right)={\sum }_{{s}_{t+1}}p\left({s}_{t+1}|{s}_{t},{a}_{t}\right)\left[c\left({a}_{t}\right)+r\left({s}_{t+1}\right)+{\gamma }_{temp}V\left({s}_{t+1},d\right)\right]$$
  2 $$V({s}_{t},d)=\left\{\begin{array}{c}0\\ \underset{{a}_{t}}{max}Q\left({s}_{t},{a}_{t},d\right)\end{array}\right.,for t>min\left(T,d\right),otherwise$$
  where $Q\left(s,a\right)$ denotes state-action values and $V\left({s}_{t},d\right)$ represents state values. Backward induction^1^ is used to compute Q-values, starting by setting $V\left({s}_{t},d\right)$ to zero when either the planning depth $d$ or the number of steps $T$ is reached. The algorithm then iterates backward to compute optimal action values at earlier steps. The term $c\left(a\right)$ represents the immediate costs of executing action $a$, while ${\gamma }_{temp}$ is the temporal discounting rate of future outcomes. Note that we considered ${\gamma }_{temp}=1$ as this parameter is not simultaneously identifiable with planning depth.
  Participants’ action choices are modeled probabilistically with a softmax function^1^ based on the computed $Q$-values from the optimal forward planning algorithm. The higher the relative value of an action, the higher the probability of selecting that action. For our case of two available actions, this corresponds to a sigmoid transformation $\sigma \left(x\right)$ of the difference between the corresponding $Q$-values, $\Delta Q\left({s}_{t},d\right)$. Choice probabilities were thus defined as:
  3 $$p\left({a}_{t}={\prime}jump{\prime}|{s}_{t},d\right)=\sigma \left(\beta *\Delta Q\left({s}_{t},d\right)+\theta \right),$$
  4 $$\sigma \left(x\right)=\frac{1}{1+{e}^{-x}}$$
  5 $$\Delta Q\left( {s_{t} ,d} \right) = Q\left( {a_{t} = \prime jump\prime ,s_{t} ,d} \right) - Q\left( {a_{t} = \prime move\prime ,s_{t} ,d} \right)$$
  where $Q\left({a}_{t},{s}_{t},d\right)$ represents the expected reward for the respective action (*jump* or *move*) at state ${s}_{t}$ and depth $d$.
  Choice probability is modified by a participant-specific inverse decision temperature (parameter beta, $\beta$) and an action bias (parameter theta, $\theta$). The inverse decision temperature $\beta$ controls the extent to which differences in $Q$-values affect action selection. If $\beta =0$, actions are selected with equal probability independent of outcomes, while higher values of $\beta$ lead to higher probabilities to select the action with the highest $Q$-value. The parameter $\theta$ denotes an a priori response bias, where positive values imply a bias towards choosing *jump*.
  The initial assumption about transition probabilities was given to the model as the true state transition probabilities. However, since we did not provide explicit information on state transition probabilities for the *jump* action $p\left({s}_{t+1}|{s}_{t},{a}_{t}={\prime}jump{\prime}\right)$, we adopted an experience-driven learning approach to let an agent adjust the corresponding state transition probabilities for both the high- and low-noise conditions. The process involves updating the belief regarding the probability of a successful *jump* at action step $t$, represented as ${\rho }_{t}=p\left({s}_{t+1}={\prime}target{\prime}|{s}_{t},{a}_{t}={\prime}jump{\prime}\right)$, using the temporal difference learning rule, which is formulated as follows:
  6 $${\rho }_{t+1}={\rho }_{t}+\alpha \left({o}_{t}-{\rho }_{t}\right)$$
  This updating mechanism is contingent upon the observed outcomes of *jump* attempts, characterized as either success $\left({o}_{t}=1\right)$ or miss $\left({o}_{t}=0\right)$. The learning rate alpha ($\alpha \in \left[\text{0,1}\right]$), is the rate at which participants adjust their assumptions regarding the probability of transition success. Larger values of $\alpha$ can also be interpreted as a faster fading of past experiences and stronger influence of more recent outcomes on decision-making. The free parameters of the full-breadth planning model are $\alpha$, $\beta$, and $\theta$.
2. *Discounted full-breadth planning:* In this model, the action sequences that include probabilistic *jump* actions are discounted. Again, participants’ action choices per mini-block are modeled in a mixture model of three model-based RL agents with the three planning depths. It differs from the full-breadth planning model in the computation of the relative action values:
  7 $$\Delta Q\left({s}_{t},d\right)={\gamma }_{prob}Q\left({a}_{t}={\prime}jump{\prime},{s}_{t},d\right)-Q\left({a}_{t}={\prime}move{\prime},{s}_{t},d\right)$$
  here, the expected reward $Q\left({a}_{t}={\prime}jump{\prime},{s}_{t},d\right)$ of the *jump* action is multiplied by a discounting factor ${\gamma }_{prob}$ to yield the subjective value of the *jump* action ${\gamma }_{prob}Q\left({a}_{t}={\prime}jump{\prime},{s}_{t},d\right)$. Consequently, in this implementation, probability discounting occurs after the computation of $Q$-values, which are still based on the optimal forward planning algorithm, consistent with the full-breadth planning model (1).
  The discounting factor follows a typical hyperbolic discounting function^14^.
  8 $${\gamma }_{prob}=\frac{1}{1+ \kappa {q}_{t+1}}$$
  9 $${q}_{t+1}= \frac{1-{\rho }_{t+1}}{{\rho }_{t+1}}$$
  The hyperbolic discounting function ${\gamma }_{prob}$ is also modified by an individual discounting parameter kappa ($\kappa$). For $\kappa =0$, ${\gamma }_{prob}Q\left({a}_{t}={\prime}jump{\prime},{s}_{t},d\right)$ equals the undiscounted expected value $Q\left({a}_{t}={\prime}jump{\prime},{s}_{t},d\right)$.
  Larger $\kappa$-values indicate stronger discounting of probabilistic outcomes, where *jump* actions leading to uncertain gains have a lower subjective value and become therefore less likely to be chosen in an action sequence, while *jump* actions leading to uncertain losses have a higher subjective value and become more likely to be chosen. The $\kappa$-values were limited at a maximum of 30, as values beyond this threshold would not offer additional information. Beliefs ${\rho }_{t+1}$ about the likelihood of the high-probability *jump* transition are again updated with an individual learning rate $\alpha$. The free parameters of the discounted full-breadth planning model are $\alpha$, $\beta$, $\theta$ and $\kappa$.
3. *Low-probability pruning:* this model is based on the hypothesis that participants prune branches of low-probability transitions and treat *jump* actions as deterministic. Again, participants’ action choices per mini-block are modeled in a mixture model of three model-based RL agents with the three planning depths. Mathematically, it differs from the full-breadth planning model in the belief about the likelihood of a high-probability *jump* at an action step $t$, ${\rho }_{t}=p\left({s}_{t+1}={\prime}target{\prime}|{s}_{t},{a}_{t}={\prime}jump{\prime}\right)=1$, which we fixed at 1. We did not expect this to change across the task, i.e. the model assumes both, the *move,* and the *jump* action, as deterministic following the given transition matrix. Hence, this model does not include a learning rate parameter $\alpha$. The computation of $Q$-values in the low-probability pruning model differs from the full-breadth planning model because it is based on a modified transition function. Specifically, $Q$-values are computed using a deterministic transition matrix in which low-probability transitions are pruned, effectively treating *jump* actions as always leading to their high-probability outcome. The free parameters of the low-probability pruning model are $\beta$ and $\theta$.
4. *Discounted low-probability pruning*: This model is based on the hypothesis that for the *jump* action, both the discounting and pruning mechanisms may be used for planning in conjunction. Again, participants’ action choices per mini-block are modeled in a mixture model of three model-based RL agents with the three planning depths. Here, action branches of low-probability transitions are pruned away, and remaining high-probability transitions are additionally discounted using their respective true probabilities. Hence, this model does not include a learning rate parameter. Consequently, $\rho$ is kept constant depending on the respective noise condition, i.e., ${\rho }_{low noise} = .9$ and ${\rho }_{high noise} = .5$ instead of ${\rho }_{t}$ in Equation (9) resulting in the corresponding odds ${q}_{low noise}$ and ${q}_{high noise}$ instead of ${q}_{t}$. The $Q$-values are calculated as in the low-probability pruning planning model. The free parameters of the discounted low-probability pruning model are $\beta$, $\theta$, and $\kappa$.
  To ensure that the experimental conditions were accurately reflected in the models, we provided all agents with information about the noise condition of each mini-block, mirroring the visual cues presented to participants during the task.

### Planning depth and parameter inference

To infer distributions over the described free model parameters and the planning depth $d$, we used approximate Bayesian inference given the hierarchical generative model. Specifically, we used a hierarchical probabilistic generative model and a hierarchical approximate posterior. As described in the previous sections, this model includes participant-specific parameters and mini-block-level information for choices and learning. Planning depth $d$ was modeled at the mini-block level, while all other model parameters were modeled at participant level. Since obtaining analytical solutions for the parameter posteriors proved intractable, we used stochastic variational inference using the Pyro v1.5.2 probabilistic programming library^38^ to infer the approximate posterior distributions. For a more detailed description of the inference procedure see Steffen et al.^12^.

For each model, first approximate posteriors of the free parameters were computed for each planning depth $d\in [1, 2, 3]$. Second, the respective inferred parameter distributions were used to infer the posterior over $d$. Note that the response likelihood is a function of a specific $d$ (10). Therefore, the response likelihood of each participant corresponds to a mixture model of these three possible, planning depth dependent, response likelihoods. Hence, we can express the mixture model for the response likelihood as a probability-weighted sum over planning depths:

10 $$p\left({a}_{b}|{s}_{b}\right)={\sum }_{d=1}^{3}p\left({d}_{b}=d\right)p\left({a}_{b}|{s}_{b},{d}_{b}=d\right)$$

These response likelihoods based on the mixture model were maximized during parameter inference.

Crucially, we assume that the majority of forward planning should occur prior to the first action within each mini-block. Consequently, when analyzing planning behavior, our main variable of interest is the planning depth before the first action. $p\left({d}_{b}=d\right)$ represents the inferred probability for planning depth $d$*.* A uniform Dirichlet prior over planning depths was used for inference.

### Statistical analyses of behavioral data

All analyses conducted for neuropsychological and behavioral data, model fit assessments, and inferred model parameters were performed using RStudio, Version 4.0.3^39^.

In the SAT, we required participants to perform beyond chance level to be included in the data analysis. This threshold (650 points) was determined based on the average fuel points accumulated by a simulated computational agent that behaved randomly ($\beta =0$). We excluded three young and seven older participants who fell below this threshold. This resulted in a final sample size of 57 young and 50 older adults. The initial 10 mini-blocks of each noise condition were excluded from all analyses as training mini-blocks, leaving 120 mini-blocks per participant for data analysis. Prior to all statistical analyses, we assessed the data distribution for normality (indicated by a non-significant Shapiro–Wilk-test’s result) and homoscedasticity (indicated by a non-significant Levene’s test result) and applied appropriate statistical tests accordingly. In all statistical tests an alpha-level of 0.05 was applied. Effect sizes are indicated by the generalized measure of partial *η*^2^.

To analyze SAT performance, we converted the total fuel points obtained during the task into a relative performance measure. We established reference points of 0% and 100% based on the average point gain of 1000 simulated full-breadth planning agents under the respective noise conditions. 0% represented the average gain of random computational agents ($\beta =0$*, *$d=3$*,* $\theta =0$), and 100% represented the average gain of optimal computational agents ($\beta =3$*, *$d=3$*,* $\theta =0$). We scaled each participant’s absolute amount of points between those reference points. Note that this can lead to participants performing above 100% if they experienced favorable random transitions for *jump*. We refer to the duration from onset of the mini-block until the first choice as planning time. Outlier analysis on the mini-block level (cut-off = 1 s) revealed no mini-blocks that needed to be excluded.

First, we determined group differences in behavioral task measures. Two-way mixed ANOVA models were utilized to assess the effects of age group as a between- and noise condition as a within-participant factor on relative task performance and planning time as the respective dependent variable. To analyze the relationship between relative SAT performance, the neuropsychological covariates, and planning time for the age groups, we performed a multiple linear regression analysis. The measure of relative performance across the entire task was regressed on age group, IDP and SWM performance, NFC score, and planning time as predictors. In an additional model we regressed relative performance across the task on age group and the free model parameters of the winning model from our model comparison (low-probability pruning model with the parameters $\beta$, $\theta$, $\kappa$, planning depth $d$) to determine the parameters’ contribution for explaining planning performance (see Supplementary Material Table S2 for a summary of the results).

### Goodness of model fit and model comparison

We compared the fits of the four models (see Model comparison) on group and noise condition level to assess differences in evidence for the respective models. We computed pseudo-Rho-squared (${\rho }^{2}$)^40^ for each model as a standardized measure of model fit as variance explained by the model. Importantly, the compared models have different numbers of free parameters: Four in the full-breadth planning model ($\alpha , \beta , \theta , d$) five in the discounted full-breadth planning ($\alpha , \beta , \theta , \kappa , d$), three in the low-probability pruning model ($\beta , \theta , d$), and four in the discounted low-probability pruning model ($\beta , \theta , \kappa , d$). Since models with more parameters tend to have a better fit than models with less parameters, we additionally computed the Bayesian Information Criterion (*BIC*)^41^ to determine the quality of model fit, adjusted for number of free model parameters with the number of observations. To quantify and interpret the strength of evidence for each model across age groups and noise conditions according to the *BIC*, we calculated the difference $BIC\Delta 1 2$ for each pair of models (see Supplementary Material, section *Goodness of model fit and model comparison* for a detailed description of the measures and procedure)^42^.

To analyze potential effects of age and noise level on the applied planning strategies, we additionally performed a similar model comparison based on $BIC\Delta 1 2$ as described before separately for each age group and noise level (see Supplementary Material Figure S2, Tables S3 and S4 for results).

### Comparison of inferred model parameters

We employed Wilcoxon’s rank sum test for non-normally distributed data and Welch’s two-sample t-test for normally distributed data to compare participant-level free model parameters. To compare mean planning depths between groups and experimental conditions at the mini-block level, we computed participant-specific mean values of planning depth for each noise condition. A two-way mixed ANOVA model (R package rstatix version 0.7.0)^43^ was implemented to assess the influence of age group (between participants) and noise condition (within participants) on planning depth.

## Results

### Behavioral measures

We first analyzed age group and noise condition effects on relative performance and planning time in the SAT. The two-way mixed ANOVA revealed that young adults show a generally higher relative performance than older adults, indicated by a significant main effect of age group on task performance (F(1,105) = 26.74, p < 0.001, ${\eta }^{2}$ = 0.20). There was no significant main effect for noise condition (F(1,105) = 2.22, p = 0.139, ${\eta }^{2}$ = 0.02). Instead, the effect of uncertainty was reversed in both groups: In the high-noise condition older adults’ performance was closer to the optimum compared to the low-noise condition, while young adults performed slightly less optimally under high noise (Fig. 5a) indicated by a significant interaction of age and noise condition (F(1,105) = 11.53, p < 0.001, ${\eta }^{2}$ = 0.10). The two-way mixed model ANOVA for the effect of age and noise condition on planning time indicated that overall planning time did not differ between age groups (F(1,105) = 0.44, p = 0.834, ${\eta }^{2}$ < 0.01). In both age groups high noise significantly reduced planning time (F(1,105) = 14.23, p < 0.001, ${\eta }^{2}$ = 0.12). There was no significant interaction effect of age group and noise condition on planning time (F(1,105) = 2.42, p = 0.123, ${\eta }^{2}$ = 0.02, Fig. 5b).

Next, we assessed the potential relationship between relative performance, age groups, cognitive covariates and planning time. A multiple linear regression analysis including age group, IDP and SWM performance, NFC score and planning time as predictors explained 49% of the variance in relative performance in the SAT (*R*^2^ = 0.491). It was found that younger age (Beta = − 0.49, p < 0.001), longer planning time (Beta = 0.44, p < 0.001), and higher SWM performance (Beta = 0.21, p = 0.016) significantly predicted higher relative performance in the SAT (see Supplementary Material Table S1 for parameter estimates).

### Model-based analyses and comparison

**Fig. 4:**
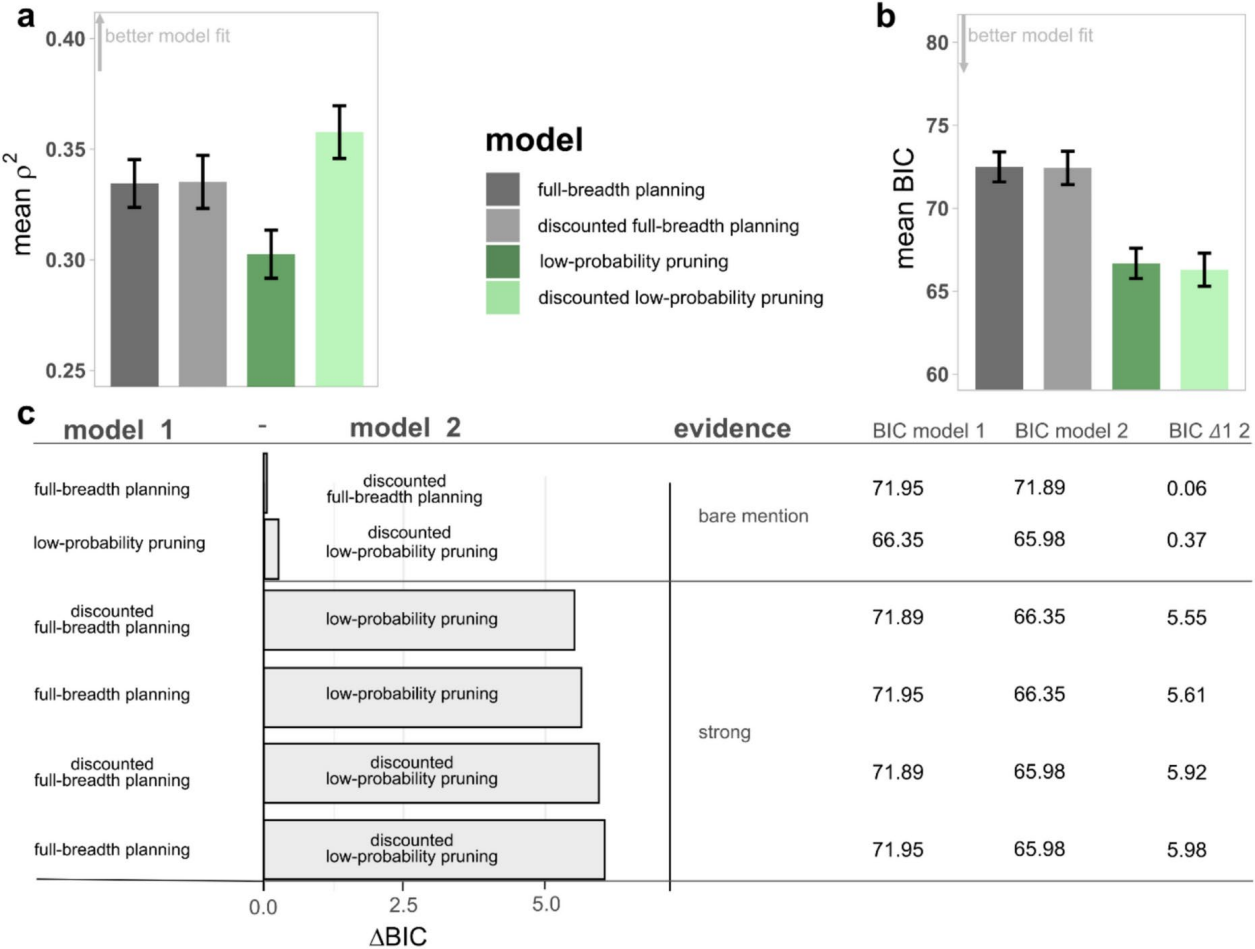
Model comparison. Model fit mean across group and condition. Error bars indicate standard error of the mean. (**a**) Pseudo Rho-squared *ρ*^2^ (standardized measure of model fit, uncorrected), higher values indicate a better model fit. (**b**) Bayesian Information Criterion ($BIC$), lower values indicate a better model fit. (**c**) Evaluation of all six possible pair-wise comparisons of model evidence using $BIC\Delta 1 2$. Absolute (rounded) values are reported for better interpretability. Model evidence interpretation is based on Neath & Cavanaugh^42^ with “bare mention” for $0 \le BIC\Delta 1 2\le 2$, “positive” for $2<BIC\Delta 1 2\le 6$, and “strong” for $6<BIC\Delta 1 2\le 10$.

Comparisons of models with different planning strategies enabled us to test whether low-probability branches of the decision tree are pruned (full-breadth planning versus low-probability pruning) and whether participants discount probabilistic rewards (no probability discounting ($k=0$) versus probability discounting). To do this, we averaged the model fit measures across age groups and noise conditions (Fig. 4) and compared differences in model fits based on ${\rho }^{2}$ as a raw measure of variance explained by the model. To account for the number of free parameters, we also compared models based on the pairwise differences of BIC ($BIC\Delta 1 2$). In the interest of interpretability, we present absolute $BIC\Delta 1 2$ values. Notably, the discounted low-probability pruning model exhibited the highest explanatory power, as indicated by the largest ${\rho }^{2}$ (Fig. 4a). When using the $BIC\Delta 1 2$ for model comparison, i.e. implicitly accounting for the number of parameters, both the undiscounted and the discounted low-probability pruning models were equivalently good models (Fig. 4b,c). In both cases, there was robust evidence against the undiscounted and the discounted full-breadth planning models, with a preference leaning towards the discounted low-probability pruning model. The result that the discounted low-probability pruning model won in the model comparison against the model with the most parameters underlines that it effectively explains the task behavior. Given the noteworthy evidence in favor of the discounted low-probability pruning model, we opted to base subsequent analyses of model parameters on this model.

In an exploratory model comparison split by age group and uncertainty condition, we did not find evidence that these factors have an effect on the application of the planning strategy, i.e., the results for each age group and noise condition generally resemble the results across those factors (see Supplementary Material Figure S2, Tables S2 and S3). We interpret this as evidence that participants of both age groups used the same planning model in both noise conditions.

### Model parameters

In this section, we present the results of the inferred parameters pertaining to the discounted low-probability pruning model, which describes a planning strategy in which participants prune low-probability branches and additionally discount the value of the remaining high-probability branch, see section *Cognitive Models*. In the model comparison (see previous section), the discounted low-probability pruning model had the lowest $BIC$ value compared to the three other models and thus the best model fit taking into account the number of parameters.

**Fig. 5:**
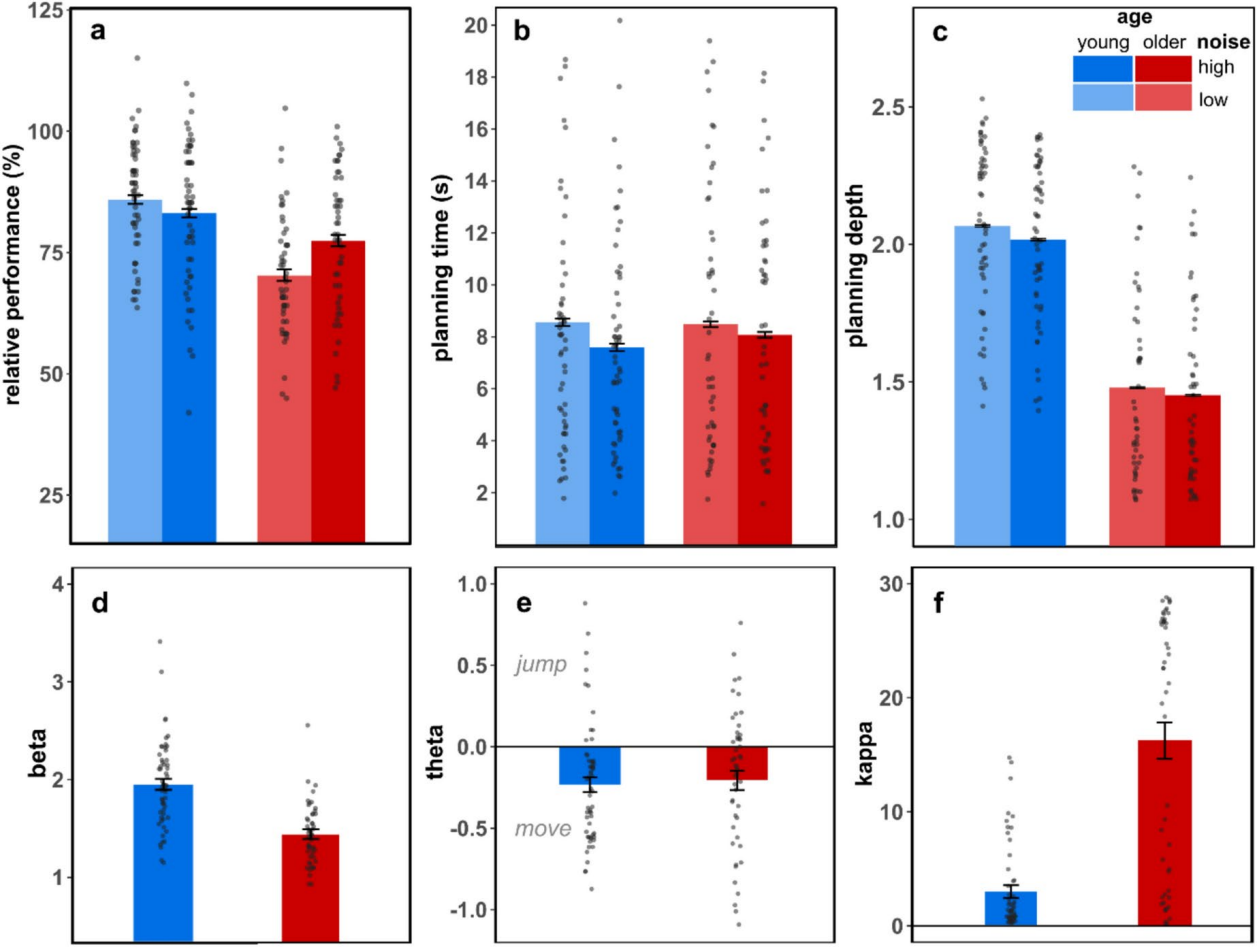
Group and condition means of behavioral measures and inferred model parameters. (**a**) Relative performance as absolute gain of points scaled between the mean gain of points of an optimal planner and a random agent. (**b**) Planning time in seconds as the interval between visual mini-block onset and execution of first action. (**c**) Planning depth: Inferred mean number of planning steps that were considered in the action plan when first action was executed. (**d**) Beta ($\beta )$: Inverse decision temperature (decision noise), larger values indicate a lower decision noise. (**e**) Theta ($\theta$): Action bias, negative values indicate a bias towards move action. (**f**) Kappa ($\kappa$): Hyperbolic discounting parameter, larger values indicate a stronger discounting of rewards reached by a probabilistic jump transition. Error bars indicate standard error of the mean. Note that the error bars are very small in (**b**) and (**c**).

For the inferred mean planning depth, a two-way mixed ANOVA revealed a significant main effect of age (F(1,105) = 92.61, p < 0.001, ${\eta }^{2}$ = 0.47), i.e., young adults planned deeper with 2.21 steps under low and 2.16 under high noise than older adults with 1.55 and 1.52 steps, respectively (Fig. 5). Both groups showed a slight but significant reduction in planning depth under high compared to low noise (F(1,105) = 63.37, p < 0.001, ${\eta }^{2}$ = 0.37; 0.05 steps in young and 0.03 steps in older adults). There was also a significant interaction of age × noise (F(1,105) = 4.72, p = 0.043, ${\eta }^{2}$ = 0.04), with young adults showing a slightly stronger reduction of planning depth under high noise compared to older adults. Deeper planning was positively correlated with relative performance (r = 0.764, p < 0.001) and planning time (r = 0.450, p < 0.001).

We found a significant age difference for the mean inverse decision temperature $\beta$, (W = 2380, p-value < 0.001), indicating higher decision noise in the group of older adults ($\beta$ = 1.43), compared to the young group ($\beta$ = 1.92). Both age groups had negative mean $\theta$ values, which differed significantly from zero (young adults: $\theta$ = − 0.21, V = 302, p < 0.001; older adults: $\theta$ = − 0.19, V = 336, p = 0.004), indicating a slight bias towards the deterministic *move* action. The bias did not differ significantly between age groups (W = 1286, p = 0.387). The mean hyperbolic discounting factor $\kappa$ was significantly higher in older adults ($\kappa$ = 15.99) compared to young adults ($\kappa$ = 2.86; W = 464, p < 0.001), indicating a substantially stronger discounting of probabilistic actions.

## Discussion

In this study, we assessed planning strategies within a three-step probabilistic decision-making task. In each mini-block, participants collected points by navigating a spaceship through a planetary constellation. For each step they could choose between a deterministic and a probabilistic action. The probabilistic action had three possible outcomes, one with a higher probability (90% or 50%) and two with a lower probability (5% or 25%). The results demonstrate that participants adopt an efficient strategy to reduce computational costs by pruning low-probability branches of the decision tree in addition to reducing the depth of planning. We show that planning is also influenced by discounting of probabilistic outcomes. In an exploratory comparison, no discernible age differences regarding the pruning of low-probability branches were found, however older adults exhibited stronger probability discounting compared to younger individuals.

With the present study we replicated our previous findings reported by Steffen et al.^12^, revealing a consistent pattern wherein young adults exhibited superior planning performance compared to older adults. Additionally, our study substantiated previously observed age differences in planning depth as a cost-reducing mechanism, with older adults demonstrating lower planning depth than their young counterparts, as well as higher response noise.

Most importantly, our study extends the existing understanding of planning in probabilistic task environments by showing that participants limit the complexity of the decision tree in our task by ignoring branches with low probability (≤ 25%) and only considering those branches with ≥ 50% probability. As our simulations demonstrate, this is a highly efficient heuristic in terms of cost-effectiveness, leading to optimal performance under low noise and 89% of optimal performance under high noise with one-eight of the effort, i.e., while ignoring almost 90% of the decision tree. Thus, participants are capable of deploying an extremely efficient heuristic for planning in a probabilistic environment, aligning with existing literature on the efficiency of resource allocation in planning processes in deterministic environments^11,44,45^ as well as the observation that humans typically find heuristics that allow good performance, while ignoring a majority of the information a full-planning agent would use^3^. However, the underlying meta-control mechanisms, i.e. processes that monitor and regulate the selection of planning strategies^46^ remain unclear based on the present data. Pruning seems to be applied based on a cost–benefit estimate made by the planner, identifying it as an efficient strategy. Nevertheless, it is still unclear how such a cost–benefit ratio can easily be estimated without high cognitive costs. Alternatively, pruning might just be reflexively driven by option probabilities, prompting future studies of whether branch probability ratios or absolute values influence pruning decisions. Future studies with an adapted task design could address these questions: Participants switching strategies when pruning leads to higher losses would support the cost–benefit estimate as a meta-control mechanism, while testing with alternating probability distributions could reveal if pruning is influenced by option probabilities.

However, the efficiency of the low-probability pruning strategy is reduced by additionally discounting probabilistic outcomes (see Fig. 3). Evidence for probability discounting has been substantiated in previous studies^13–15^, and we now provide evidence for this bias in a multi-step probabilistic planning task.

Despite applying the same discounted low-probability pruning strategy under low and high noise, participants exhibited a decrease in depth of planning and planning time under high noise. Given that the decision trees had equal complexity in both conditions, the diminished planning time suggests a reduction of cognitive effort^47^ before the initial action of high-noise relative to low-noise mini blocks. In the event of a low-probability transition, the low-probability pruning strategy requires replanning, as this scenario has not yet been considered. Given that low-probability outcomes are five times more likely to occur under high noise conditions than under low noise conditions, the necessity for replanning will increase in direct proportion. To limit total resource expenditure for a mini-block given the eventual need for replanning, participants may have therefore initially reduced the planning effort during the high noise condition—resulting in the observed reduction of planning time and depth in the high noise condition. Indeed, a post-hoc comparison of planning times corroborates this interpretation: it shows that planning time was significantly shorter when the first action was a probabilistic *jump* compared to a deterministic *move* and that this difference was larger under high compared to low noise (see Supplementary Material Table S6). In line with the hypothesis that planning depth is reduced in response to larger uncertainty, we found that individuals who more steeply discount probabilistic rewards (i.e., exhibit higher $\kappa$) also adopt shallower planning depths, potentially to minimize the effort associated with potential replanning due to uncertain state transitions (see Supplementary Material, Table S2).

Our comparison of age groups suggests that low-probability pruning is applied by both, young and older adults. Age differences in the inferred planning depth persisted even under the pruning model assumption. In other studies it has been found that heuristics are combined by the planner^6^. In line with this, we found that participants reduced their planning depth as an additional strategy to pruning low-probability branches as both age groups did not plan all of the three possible steps ahead. However, the reduction of planning depth was stronger in the older group, which might be adaptive due to more limited cognitive resources. In line with that, we further replicated from our previous study^12^ that older adults show substantially lower performances in the fluid intelligence measure of SWM. We found that this measure significantly contributed to explaining planning performance.

In addition to reduced planning depth and increased decision noise, a novel finding for the group of older adults is their stronger discounting of probabilistic outcomes compared to younger individuals. We observed a high mean discounting rate ($\kappa$) of 16 among older adults. This implies that older individuals discount an exemplary gain or loss of 100 points with 90% probability to a value of 36 points, and for a 50% probability gain or loss, it would be discounted to 6 points. The young group exhibited a mean $\kappa$ of 3, where 100 points would be discounted to 75 points or 25 points respectively. The notably high discount factors observed in our study, compared to those usually found in single-choice tasks^48^, likely stem from the multi-step nature of the task. Participants made decisions across multiple planning steps (depending on their planning depth), each involving choices between probabilistic and deterministic outcomes. Each decision influenced subsequent available options and—depending on the number of probabilistic transitions within the sequence—the probabilities of reaching the planned final goal state. Within the planning phase, this might increase the perceived uncertainty (i.e., the subjective estimation of the odds against receiving the expected reward from the planned sequence) as actual outcomes of probabilistic transitions are still unknown, observable as a multiplied discount factor compared to a single-choice task.

## Limitations

An inherent design limitation in our task was revealed through post-hoc simulations, demonstrating that participants could accumulate approximately 37% more points in the low-noise condition compared to the high-noise condition. This imbalance made direct inter-condition comparisons using absolute points unfeasible. To overcome this challenge, we introduced the relative performance measure, expressed as a percentage of optimal performance relative to random performance. However, relative performance must still be considered in connection with the planning depth. Post-hoc simulations showed that the difference of points between both conditions is not a linear function of the planning depth. Despite relative performance balancing at planning depth three, differences between high- and low-noise conditions remain noticeable at lower depths. In our behavioral data the effect of the noise condition (low/high) on relative performance appeared to be reversed in both groups, with older adults showing improved performance under high noise. Given the average planning depth of older participants (between 1 and 2), this relative improvement in high noise is likely to be attributed to the task design rather than an interpretable effect.

Notably, we observed generally worse model fits in the group of older adults. The inverse temperature parameter, accounting for response noise, probably explains discrepancies between the model and the data, as low inverse decision temperature values correspond to more random behavior and therefore lead to a lower model fit^49^. Alternatively, lower model fit in older adults may also suggest that they employed a planning strategy that we did not consider.

Our study was conducted in an unsupervised online setting, where participants completed the task presumably under diverse conditions. Our lack of control over these factors may introduce unexplained variance in the data, independent of our experimental manipulations.

## Conclusion

The findings of our study indicate that in probabilistic environments, planners are able to efficiently reduce the complexity of decision trees by pruning branches that occur with low probability. Furthermore, individuals reduce planning complexity by truncating remaining branches of the decision tree, i.e. reducing the depth of planning. Although not cost efficient, probabilistic branches seem to be discounted in the planning process. The performance of older participants was found to be lower than that of younger individuals. This may be attributed to a reduction in planning depth, an increase in decision noise, and a tendency to discount of probabilistic outcomes more steeply.

Future research should aim to improve our understanding of the mechanisms that determine the selection of a particular planning strategy: How are heuristics formed and how are they evaluated by planners in terms of their efficiency, especially with little prior experience in a given task environment? The meta-control of such arbitrations could be driven by various mechanisms such as previous experience in related contexts, cost–benefit estimates or a comparison with an average reward expectation derived from the current task context. It would also be highly relevant to better understand individual differences in the strategies used in multi-step planning and the related meta-control mechanisms, or why some people are unable to form or apply adequate heuristics. This could provide further insights into how they relate to aging or mental disorders.

## Supplementary Information

Supplementary Information.

## Data Availability

All data generated or analyzed during this study along with the code and scripts necessary to perform the model-based inference and statistical analyses are available in the GitHub repository https://github.com/SophiaHelenSass/SAT_PD2_age.
