# Supplementary material for: Heuristic pruning of decision trees at low probabilities and probability discounting in sequential planning in young and older adults
Source: Sci Rep. 2025 May 9;15:16260. doi: 10.1038/s41598-025-00905-7 (PMC12064811; doi:10.1038/s41598-025-00905-7)
Supplement: Supplementary file 1 — Supplementary Information. [file 41598_2025_905_MOESM1_ESM.pdf]

Supplementary Material

Probabilistic multi-step planning task: Debriefing questionnaire

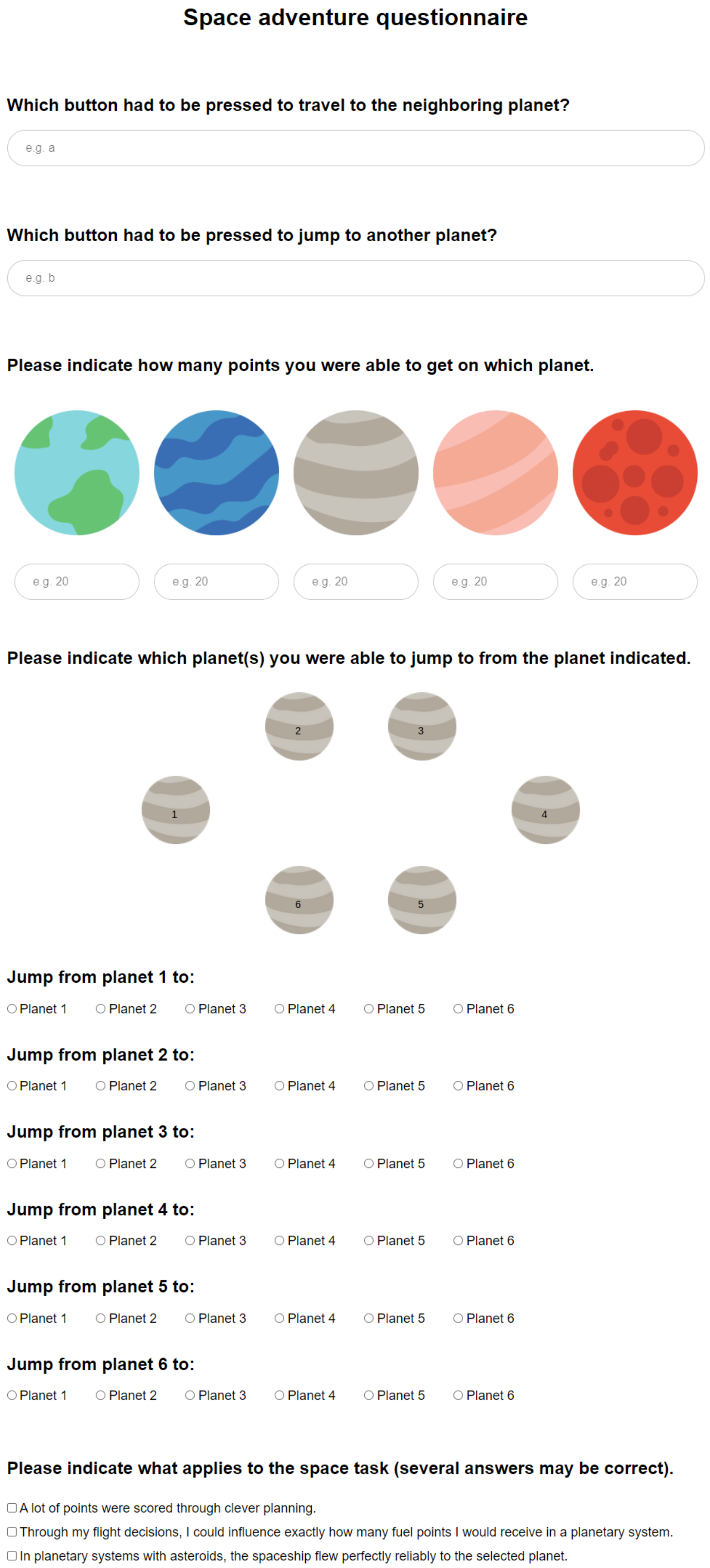

Space adventure questionnaire

Which button had to be pressed to travel to the neighboring planet?

e.g. a

Which button had to be pressed to jump to another planet?

e.g. b

Please indicate how many points you were able to get on which planet.

e.g. 20 | e.g. 20 | e.g. 20 | e.g. 20 | e.g. 20

Please indicate which planet(s) you were able to jump to from the planet indicated.

Jump from planet 1 to:

○ Planet 1 ○ Planet 2 ○ Planet 3 ○ Planet 4 ○ Planet 5 ○ Planet 6

Jump from planet 2 to:

○ Planet 1 ○ Planet 2 ○ Planet 3 ○ Planet 4 ○ Planet 5 ○ Planet 6

Jump from planet 3 to:

○ Planet 1 ○ Planet 2 ○ Planet 3 ○ Planet 4 ○ Planet 5 ○ Planet 6

Jump from planet 4 to:

○ Planet 1 ○ Planet 2 ○ Planet 3 ○ Planet 4 ○ Planet 5 ○ Planet 6

Jump from planet 5 to:

○ Planet 1 ○ Planet 2 ○ Planet 3 ○ Planet 4 ○ Planet 5 ○ Planet 6

Jump from planet 6 to:

○ Planet 1 ○ Planet 2 ○ Planet 3 ○ Planet 4 ○ Planet 5 ○ Planet 6

Please indicate what applies to the space task (several answers may be correct).

☐ A lot of points were scored through clever planning.
☐ Through my flight decisions, I could influence exactly how many fuel points I would receive in a planetary system.
☐ In planetary systems with asteroids, the spaceship flew perfectly reliably to the selected planet.

Figure S 1: Debriefing Questionnaire. Participants filled out this questionnaire after completing the Space Adventure Task to check their understanding of basic task rules. The original questionnaire was presented in German.

Multiple linear regression analyses: cognitive covariates and relative performance in the SAT

Predictor	Unstandardized Coefficients		Standardized Coefficients	t	p	95% CI b	
	b	*SE*	*Beta*			*LL*	*UL*
Intercept	57.62	10.75	-	5.36	**<.001**	36.28	78.96
Age group	-12.06	2.81	-0.49	-4.29	**<.001**	-17.65	-6.48
IDP accuracy (%)	-0.10	0.09	-0.12	-1.08	.283	-0.29	0.08
SWM accuracy (%)	0.27	0.11	0.21	2.44	**.016**	0.05	0.48
NFC score	0.07	0.07	0.07	0.94	.348	-0.07	0.21
SAT planning time (s)	1.19	0.20	0.44	6.05	**<.001**	0.80	1.58

Table S 1. Linear Regression Analysis including age group, IDP performance, SWM performance, NFC score and planning time as predictors for SAT relative performance. Age group reference category: young adults. CI = confidence interval; *LL* = lower limit; *UL* = upper limit. Model Summary: R^2 =.491; Adj. R^2 = .466; *SE* = 9.02

Multiple linear regression analyses: inferred model parameters and relative performance in the SAT

Predictor	Unstandardized Coefficients		Standardized Coefficients	t	p	95% CI b	
	b	*SE*	*Beta*			*LL*	*UL*
Intercept	31.92	6.91	-	4.62	<.001	17.06	43.51
Age group	4.74	2.20	0.19	2.16	**.033**	0.39	9.10
d	17.06	2.89	0.66	5.91	**<.001**	11.33	22.79
β	9.15	2.03	0.34	4.51	**<.001**	5.12	13.17
θ	0.88	2.12	0.03	0.41	.681	-3.33	5.08
κ	-0.08	0.11	-0.07	-0.70	.488	-0.31	0.15

Table S 2. Linear Regression Analysis including age group and discounted low-probability pruning model parameters β, θ, κ, and planning depth d as predictors for SAT relative performance. Age group reference category: young adults. CI = confidence interval; *LL* = lower limit; *UL* = upper limit. Model Summary: R^2 = .657; Adj. R^2 = .640; *SE* = 7.406

We found that the hyperbolic discounting parameter κ was not a significant predictor of relative performance in the SAT. This result is likely influenced by collinearity effects, as κ was found to be highly correlated with planning depth d (r = -.772, p < .001).

A plausible theoretical explanation for this negative association is that individuals who more steeply discount probabilistic rewards (i.e., exhibit higher κ) overestimate the uncertainty of those rewards in the planning process. Consequently, they may adopt shallower planning depths to minimize the effort associated with potential replanning due to uncertain state transitions.

Goodness of model fit and model comparison

We compared the fits of four RL models averaged across age groups and noise condition level to assess differences in evidence for the respective models. As a raw measure of model fit, we first computed the negative log-likelihood $l(\phi)$ (NLL, Equation (S 1) for each model. The NLL denotes the log-likelihood of participants' action choices $\{a_t\}$ given the inferred set of parameters summarized as ϕ.

$$\boldsymbol{l(\phi) = -\log P_{\phi}\left[\{a_t\}_{t=1}^{N}\right] = -\sum_{t=1}^{N} \log p_t(a_t/\phi)} \qquad \text{(S 1)}$$

Choice probabilities $p_t(a_t|\phi)$ denote the average of individual choice probabilities per planning depth d, weighted by the probability inferred for each planning depth per mini-block. Based on the NLL we then computed pseudo-Rho-squared (ρ^2) [1] for each model as a standardized measure of model fit as variance explained by the model (Equation (S 2)).

$$\rho^2 = 1 - \frac{l(\phi)}{l_{random}} \qquad \text{(S 2)}$$

The compared models have different numbers of free parameters: Four in the full-breadth planning model (α, β, θ, d), five in the discounted full-breadth planning model ($\alpha, \beta, \theta, \kappa, d$), three in the low-probability pruning model (β, θ, d), and four in the discounted low-probability pruning model (β, θ, κ, d). Since models with many parameters tend to have a better fit than models with less parameters, we additionally computed the Bayesian Information Criterion (BIC) [2] to determine the quality of model fit, adjusted for number of free model parameters m with the number of observations n (Equation (S 3)).

$$BIC = 2l(\hat{\phi}) + m \log n \qquad \text{(S 3)}$$

To quantify and interpret the strength of evidence for each model across age groups and noise conditions according to the BIC, we calculated the difference $BIC\Delta 1\ 2$ (Equation (S 4)) for each pair of models, where $k1$ and $k2$ represent two out of the four models being compared [3]. Model evidence interpretation is based on Neath &

Cavanaugh (2012) with “bare mention” for $0 \leq BIC\Delta 1\,2 \leq 2$, “positive” for $2 < BIC\Delta 1\,2 \leq 6$, “strong” for $6 < BIC\Delta 1\,2 \leq 10$, , and “very strong” for $BIC\Delta 1\,2 > 10$.

$$BIC\Delta 1\,2 = BIC(k1) - BIC(k2) \tag{S 4}$$

To explore potential effects of age group and noise level on the applied planning strategy, we additionally computed ρ^2 and BIC for the respective age groups and noise condition level. We compared the group- and condition-specific model fits using $BIC\Delta 1\,2$ (Equation (S 4)). The results for the comparison of model fits split by age group and noise condition are reported in the next section.

Model comparison split by age group and noise condition

For completeness, we provide the results of the model comparison of the four behavioral models (full-breadth planning, discounted full-breadth planning, low-probability pruning, discounted low-probability pruning) split by age group and noise condition to explore potential effects of those factors on the low-probability pruning strategy and the discounting of probabilistic rewards. Mean values of pseudo Rho-squared ρ^2 and BIC for each age group and noise condition are depicted in Figure S 2. The pairwise comparison using $\Delta BIC\ 1\ 2$ is reported in Table S 3 and Table S 4. Notably, the discounted low-probability pruning model exhibited the highest explanatory power for all groups and noise conditions, as indicated by the largest ρ^2 (Figure S 2 a). This is further corroborated when accounting for the number of parameters (BIC). Only for young adults under high noise the low-probability pruning model without the discounting bias showed a better fit in the BIC than the discounted one, while both models explain an equal amount of variance.

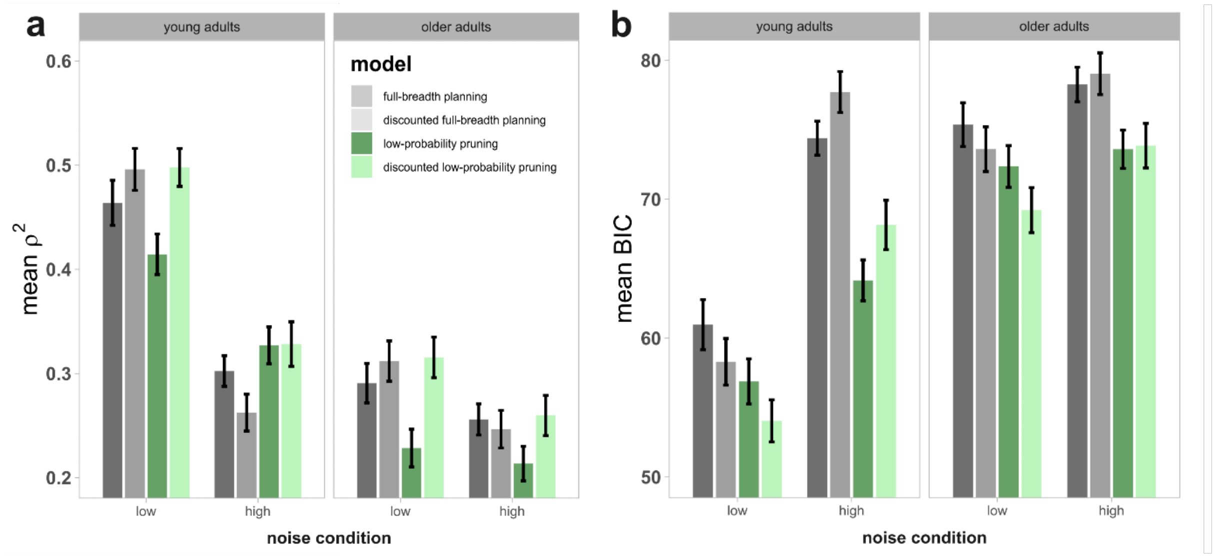

Figure S 2: Model fit and model comparison measures split by condition and age group. Values are shown per age group and noise condition. **a** mean pseudo Rho-squared ρ^2 (standardized measure of model fit, uncorrected) of the respective RL model. Larger values indicate a better model fit. **b** Bayesian Information Criterion ($\boldsymbol{BIC}$; corrected for the number of free parameters in the model) of the respective RL model. Lower values indicate a better model fit. Error bars indicate standard error of the mean.

**Young adults**					
model $k1$	model $k2$	evidence	$BIC\ k1$	$BIC\ k2$	$\Delta BIC1\ 2$
**Low noise (low-probability transition p = 0.05)**					
discounted full-breadth planning	full-breadth planning	positive	58.30	60.97	2.67
low-probability pruning	discounted low-probability pruning	positive	56.89	54.05	2.84
full-breadth planning	low-probability pruning	positive	60.97	56.89	4.08
discounted full-breadth planning	low-probability pruning	bare mention	58.30	56.89	1.41
full-breadth planning	discounted low-probability pruning	strong	60.97	54.05	6.92
discounted full-breadth planning	discounted low-probability pruning	positive	58.30	54.05	4.25
**High noise (low-probability transition p = 0.25)**					
discounted low-probability pruning	low-probability pruning	positive	68.15	64.16	3.99
discounted full-breadth planning	full-breadth planning	positive	77.72	74.40	3.32
full-breadth planning	discounted low-probability pruning	strong	74.40	68.15	6.25
full-breadth planning	low-probability pruning	very strong	74.40	64.16	10.24
discounted full-breadth planning	discounted low-probability pruning	strong	77.72	68.15	9.57
discounted full-breadth planning	low-probability pruning	very strong	77.72	64.16	13.56

Table S 3: Model comparison split by condition for young adults. Comparison based on Bayesian Information Criterion (BIC). Evaluation of model evidence based on the measure of $BIC\Delta 1\ 2$ [3]. Entries for each noise condition sorted by increasing $BIC\Delta 1\ 2$ values. Evaluation of all six possible pair-wise comparisons of model evidence using $BIC\Delta 1\ 2$. Absolute (rounded) values are reported for better interpretability. Model evidence interpretation is based on Neath & Cavanaugh (2012) with “bare mention” for $0\ \leq BIC\Delta 1\ 2 \leq 2$, “positive” for $2 < BIC\Delta 1\ 2 \leq 6$, “strong” for $6 < BIC\Delta 1\ 2 \leq 10$, , and “very strong” for $BIC\Delta 1\ 2 > 10$.

**Older adults**					
model $k1$	model $k2$	evidence	$BIC\ k1$	$BIC\ k2$	$\Delta BIC1\ 2$
**Low noise (low-probability transition p = 0.05)**					
full-breadth planning	discounted full-breadth planning	bare mention	75.37	73.61	1.77
discounted full-breadth planning	low-probability pruning	bare mention	73.61	72.36	1.25
full-breadth planning	low-probability pruning	positive	75.37	72.36	3.01
low-probability pruning	discounted low-probability pruning	positive	72.36	69.22	3.14
discounted full-breadth planning	discounted low-probability pruning	positive	73.61	69.22	4.39
full-breadth planning	discounted low-probability pruning	strong	75.37	69.22	6.15
**High noise (low-probability transition p = 0.25)**					
discounted full-breadth planning	full-breadth planning	bare mention	79.04	78.27	0.77
low-probability pruning	discounted low-probability pruning	bare mention	73.60	73.86	0.26
full-breadth planning	low-probability pruning	positive	78.27	73.60	4.67
discounted full-breadth planning	low-probability pruning	positive	79.04	73.60	5.44
full-breadth planning	discounted low-probability pruning	positive	78.27	73.86	4.41
discounted full-breadth planning	discounted low-probability pruning	positive	79.04	73.86	5.18

Table S 4: Model comparison split by condition for older adults. Comparison based on Bayesian Information Criterion (BIC). Evaluation of model evidence based on the measure of $BIC\Delta 1\ 2$ [3]. Entries for each noise condition sorted by increasing $BIC\Delta 1\ 2$ values. Evaluation of all six possible pair-wise comparisons of model evidence using $BIC\Delta 1\ 2$. Absolute (rounded) values are reported for better interpretability. Model evidence interpretation is based on Neath & Cavanaugh (2012) with “bare mention” for $0\ \leq BIC\Delta 1\ 2 \leq 2$, “positive” for $2 < BIC\Delta 1\ 2 \leq 6$, “strong” for $6 < BIC\Delta 1\ 2 \leq 10$, , and “very strong” for $BIC\Delta 1\ 2 > 10$

Proportion of best-fitting model in sample

As detailed in the manuscript, the discounted low-probability pruning model exhibited superior fit across various groups and noise levels. While the model comparison in the main text provides average fit metrics per group and noise condition, it did not specify how frequently each model was identified as the best-fitting for individual participants based on the BIC. Therefore, Figure S 3 illustrates the proportion of participants for whom each model was the best fit.

At the individual level, the discounted low-probability pruning model demonstrated the highest proportion of best fits under the low noise condition. Although this model also frequently achieved the best fit under the high noise condition, an even larger proportion of the sample was best fit by the undiscounted low-probability pruning model in that scenario. Conversely, the full-breadth planning model and its discounted variant were the best-fitting models for only a very small minority of participants.

The mean distance $\Delta BIC1\ 2$ between the best-fitting and second-best-fitting model across age groups was 2.9 (SD = 2.34, positive evidence) for the low noise condition and 5.26 (SD=3.50, strong evidence) for the high noise condition.

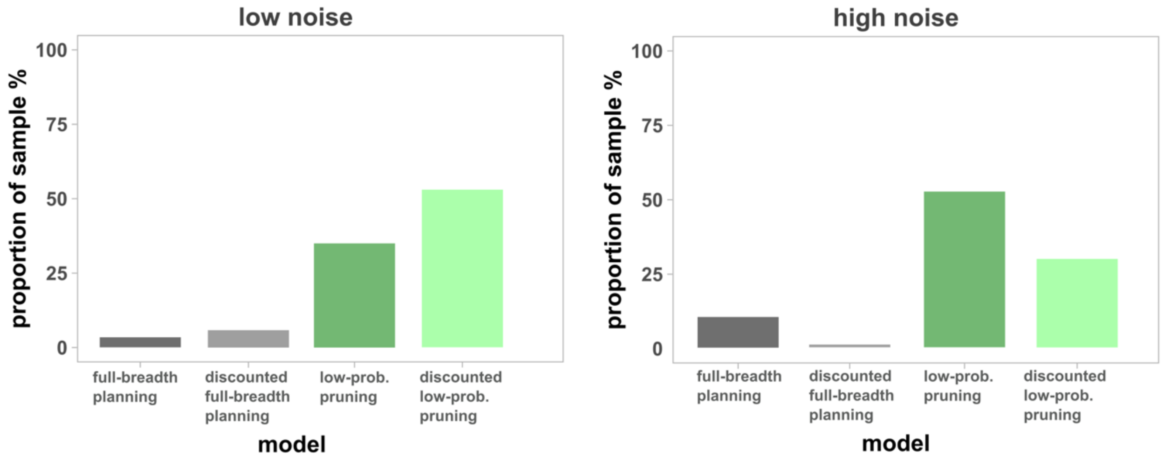

Figure S 3: Proportion of best-fitting model in sample. Data is shown in % averaged across the groups of young and older adults.

Model cross-fitting

In our study, we employed the following model cross-fitting procedure to evaulate the accuracy of our model selection. We generated a set of simulated data consisting of 100 samples using our four candidate models: full-breadth planning, discounted full-breadth planning, low-probability pruning, and discounted low-probability pruning. The respective parameters for each model were set as follows: $\alpha = 0$, $\beta = 3$, $\theta = 0$, $\kappa = 10$. Subsequently, we fitted the simulated data to each of the four alternative models, performing 30 runs with 500 iterations for each fit. To determine the best-fitting model among these alternatives, we employed the BIC and ρ^2 for comparison. For each of the two noise conditions, we selected the model exhibiting the lowest BIC and the highest ρ^2 as the best-fitting model. We generated confusion matrices and inversion matrices. These matrices quantified the probability of correctly identifying the true underlying planning model from the set of alternative models (confusion matrix) and the probability of the identified best-fitting model being the true underlying planning model (inversion matrix). This approach enabled us to assess our approach's feasibility to differentiate and reliably identify planning processes within the human data. A summary of the results can be found in Figure S 4 and Figure S 5. The probabilities shown in the figures are rounded for clarity, which may result in row sums that deviate slightly from 1.0 (100%).

The results show that agent behavior coming from different underlying models can be differentiated and identified in our model comparison, indicated by the higher probabilities along the diagonal of the matrices. Under high noise the discounted low-probability pruning model has more false positive identifications as best-fitting model based on BIC and ρ^2 than the other model alternatives (last column of the matices b and d).

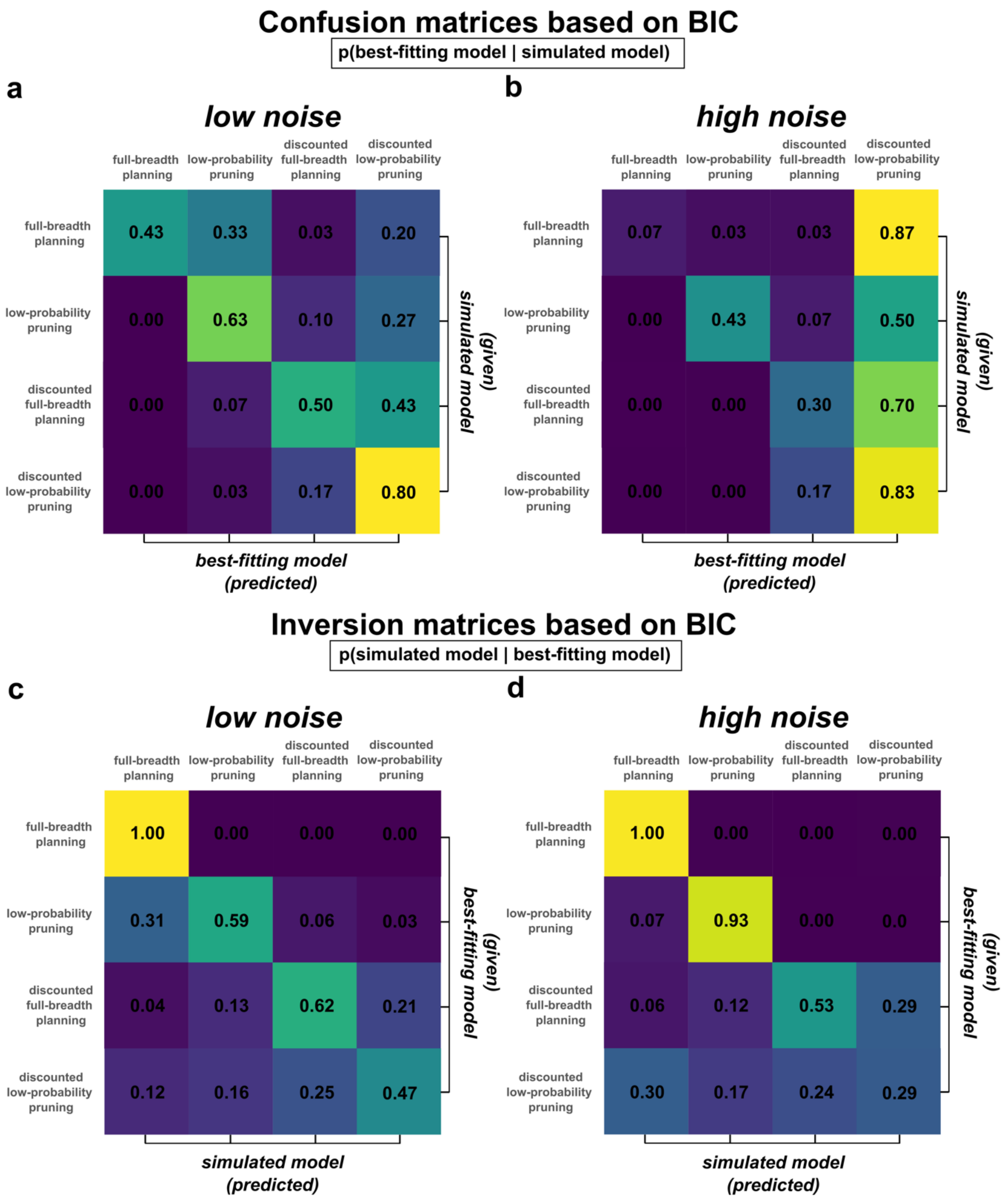

Figure S 4: Cross-fitting for model selection based on BIC. To assess the accuracy of our model selection, confusion and inversion matrices were generated. Diagonals from top left to bottom right indicate the true positives. **a-b** Confusion matrices for model selection under the task condition of low (a) and high (b) noise as the probability of a model being identified as best-fitting based on the BIC, given the simulated data from the respective model. **c-d** Inversion matrices for model selection under the task condition of low (c) and high (d) noise as the probability of the simulated data being based on a respective model, given that it was identified as the best-fitting model based on the BIC.

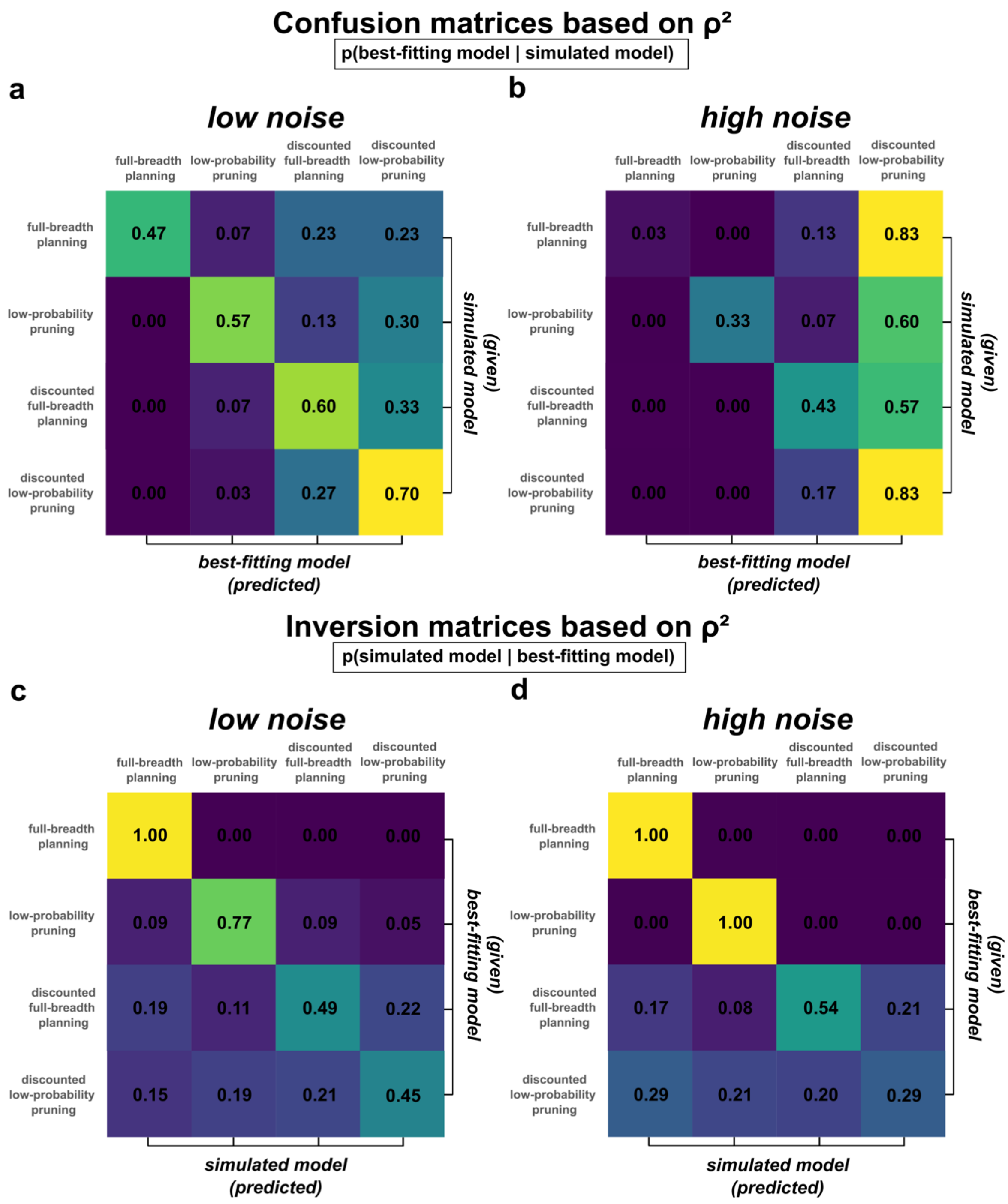

Figure S 5: Cross-fitting for model selection based on ρ^2***.*** To assess the accuracy of our model selection, confusion and inversion matrices were generated. Diagonals from top left to bottom right indicate the true positives. **a-b** Confusion matrices for model selection under the task condition of low (a) and high (b) noise as the probability of a model being identified as best-fitting based on the ρ^2, given the simulated data from the respective model. **c-d** Inversion matrices for model selection under the task condition of low (c) and high (d) noise as the probability of the simulated data being based on a respective model, given that it was identified as the best-fitting model based on the ρ^2.

Post-hoc analyses of planning times

Action choice / Age group	Low noise condition: Deterministic *move*	Low noise condition: Probabilistic *jump*	High noise condition: Deterministic *move*	High noise condition: Probabilistic *jump*
Young adults	9.15 (5.89)	8.27 (5.01)	8.46 (4.82)	7.18 (3.81)
Older adults	8.66 (5.25)	8.50 (5.26)	8.89 (5.08)	7.70 (4.27)

Table S 5: Descriptive statistics of planning time (seconds) split by age group, noise condition and action choice. Scores represent means and standard deviations (in parenthesis).

Effect	Test-statistic	p	η^2 [a]
Intercept	333.756	<.001	.74
Age group	0.04	.847	<.01
Noise condition	10.51	**.002**	<.01
Action	19.57	**<.001**	.01
Age group × noise condition	2.88	**.093**	<.01
Age group × action	1.07	.304	<.01
Noise condition × action	8.63	**.004**	<.01
Age group × noise condition × action	1.67	.199	<.01

Table S 6: Results of the mixed analysis of variance to compare planning times for deterministic and probabilistic first actions. Test statistic represents F-scores. [a] effect size partial η^2.

Participants in both age groups reduced planning depth and time under the condition of high compared to low noise, indicating a potential motivational difference. Despite similar decision tree complexity, this reduction could indicate an adaptation to the increased likelihood of low-probability outcomes under high noise. To conserve resources and minimize the eventual need for replanning, participants might reduce planning depth, resulting in shorter planning time. Thus, if the first action choice was probabilistic (*jump*), planning time should be shorter compared to a deterministic first action (*move*). A post-hoc analysis of the effects of action choice, noise condition, and age group on planning time in a three-way mixed ANOVA model corroborated this interpretation with a significant interaction of noise condition and action choice (Table

S 6, $F(1, 105) = 8.63$, $p < .001$, $\eta^2 = .001$). The reduction of planning time was consistent across age groups, indicated by a non-significant interaction effect of action choice, noise condition and age group ($F = 1.67$, $p = .199$, $\eta^2 < .001$).

Linear mixed model analyses: controlling for education level differences in planning task measures

Younger adults differed from the older group in the proportion of participants with a higher education level (i.e., ≥ 12 years of schooling; 89.47% vs. 72.00%; $\chi^2(1) = 5.35$, p = .021). To account for these differences, we conducted additional linear mixed model analyses to control for education level when examining relative task performance, planning time, and planning depth. Age group (young, older), education level (lower, higher), and noise condition (low, high) were included as fixed factors, with participants modeled as a random intercept. We tested the interaction between age group and education level to determine whether the effect of age group on planning performance varied as a function of education level. After controlling for education level, the effects of age group on the three planning measures remained unchanged. No significant interaction between age group and education level was found.

Planning task performance

Predictor	Unstandardized Coefficients		t	p	95% CI b	
	b	*SE*			*LL*	*UL*
Intercept	84.50	5.48	15.43	<.001	73.92	95.09
Age group	-19.23	6.54	-2.94	**.004**	-31.88	-6.58
Education group	1.57	5.79	0.27	.786	-9.62	12.76
Noise condition	-3.12	6.14	-0.51	.612	-15.04	8.80
Age group × education level	4.84	7.17	0.68	.500	-9.01	18.69
Age group × noise condition	15.27	7.34	2.08	**.040**	1.02	29.52
Education level × noise condition	0.38	6.49	0.06	.953	-12.22	12.98
Age group × education level × noise condition	-7.35	8.04	-0.91	.362	-22.95	8.25

Table S 7. Linear Regression Analysis including age group, noise condition and education level as predictors for SAT relative performance. Education level included as a factor with two levels, "higher (≥12 years of school education)" or "lower (< 12 years)". CI = confidence interval; *LL* = lower limit; *UL* = upper limit. Model Summary: $R^2 conditional$ = .484, $R^2 marginal$= .181, SE = 10.64

Planning time

Predictor	Unstandardized Coefficients		t	p	95% CI b	
	b	*SE*			*LL*	*UL*
Intercept	7.45	1.93	3.86	<.001	3.71	11.18
Age group	-0.54	2.30	-0.23	.815	-5.00	3.93
Education group	1.25	2.04	0.61	.542	-2.70	5.20
Noise condition	-0.82	0.77	-1.06	.292	-2.31	0.68
Age group × education level	0.92	2.52	0.37	.716	-3.97	5.81
Age group × noise condition	0.80	0.92	0.88	.383	-0.98	2.59
Education level × noise condition	-0.17	0.81	-0.21	.835	-1.75	1.41
Age group × education level × noise condition	-0.37	1.01	-0.37	.711	-2.33	1.58

Table S 8. Linear Regression Analysis including age group, noise condition and education level as predictors for SAT planning time. Education level included as a factor with two levels, “higher (≥12 years of school education)” or “lower (< 12 years)”. CI = confidence interval; *LL* = lower limit; *UL* = upper limit. Model Summary: $R^2 conditional$ = .922, $R^2 marginal$= .025, SE = 1.33

Planning depth

Predictor	Unstandardized Coefficients		t	p	95% CI b	
	b	*SE*			*LL*	*UL*
Intercept	2.20	0.14	15.52	<.001	1.93	2.48
Age group	-0.81	0.17	-4.80	**<.001**	-1.14	-0.48
Education group	0.01	0.15	0.08	.938	-0.28	0.30
Noise condition	-0.06	0.02	-2.62	**.010**	-0.11	-0.02
Age group × education level	0.21	0.19	1.14	.258	-0.15	0.57
Age group × noise condition	0.05	0.03	1.62	.108	-0.01	0.10
Education level × noise condition	0.01	0.02	0.28	.781	-0.04	0.06
Age group × education level × noise condition	-0.03	0.03	-0.91	.365	-0.09	0.03

Table S 9. Linear Regression Analysis including age group, noise condition and education level as predictors for SAT planning depth. Education level included as a factor with two levels, “higher (≥12 years of school education)” or “lower (< 12 years)”. CI = confidence interval; *LL* = lower limit; *UL* = upper limit. Model Summary: $R^2 conditional$ = .993, $R^2 marginal$= .479, SE = 0.041

References

1. Simon, D. A. & Daw, N. D. Neural Correlates of Forward Planning in a Spatial Decision Task in Humans. *J. Neurosci.* **31**, 5526–5539 (2011).
2. Schwarz, G. Estimating the Dimension of a Model. *Ann. Stat.* **6**, (1978).
3. Neath, A. A. & Cavanaugh, J. E. The Bayesian information criterion: background, derivation, and applications. *WIREs Comput. Stat.* **4**, 199–203 (2012).
